# Photonic Activation of Plasminogen Induced by Low Dose UVB

**DOI:** 10.1371/journal.pone.0116737

**Published:** 2015-01-30

**Authors:** Manuel Correia, Torben Snabe, Viruthachalam Thiagarajan, Steffen Bjørn Petersen, Sara R. R. Campos, António M. Baptista, Maria Teresa Neves-Petersen

**Affiliations:** 1 Department of Physics and Nanotechnology, Aalborg University, Aalborg, Denmark; 2 BioPhotonics Group, Department of Nanomedicine, International Iberian Nanotechnology Laboratory (INL), Braga, Portugal; 3 School of Chemistry, Bharathidasan University, Tiruchirappalli, India; 4 Department of Health Science and Technology, Aalborg University, Aalborg, Denmark; 5 The Institute for Lasers, Photonics and Biophotonics; University at Buffalo, The State University of New York, New York, United States of America; 6 Instituto de Tecnologia Química e Biológica António Xavier, Universidade Nova de Lisboa, Oeiras, Portugal; Russian Academy of Sciences, Institute for Biological Instrumentation, RUSSIAN FEDERATION

## Abstract

Activation of plasminogen to its active form plasmin is essential for several key mechanisms, including the dissolution of blood clots. Activation occurs naturally via enzymatic proteolysis. We report that activation can be achieved with 280 nm light. A 2.6 fold increase in proteolytic activity was observed after 10 min illumination of human plasminogen. Irradiance levels used are in the same order of magnitude of the UVB solar irradiance. Activation is correlated with light induced disruption of disulphide bridges upon UVB excitation of the aromatic residues and with the formation of photochemical products, e.g. dityrosine and N-formylkynurenine. Most of the protein fold is maintained after 10 min illumination since no major changes are observed in the near-UV CD spectrum. Far-UV CD shows loss of secondary structure after illumination (33.4% signal loss at 206 nm). Thermal unfolding CD studies show that plasminogen retains a native like cooperative transition at ~70 ºC after UV-illumination. We propose that UVB activation of plasminogen occurs upon photo-cleavage of a functional allosteric disulphide bond, Cys737-Cys765, located in the catalytic domain and in van der Waals contact with Trp761 (4.3 Å). Such proximity makes its disruption very likely, which may occur upon electron transfer from excited Trp761. Reduction of Cys737-Cys765 will result in likely conformational changes in the catalytic site. Molecular dynamics simulations reveal that reduction of Cys737-Cys765 in plasminogen leads to an increase of the fluctuations of loop 760–765, the S1-entrance frame located close to the active site. These fluctuations affect the range of solvent exposure of the catalytic triad, particularly of Asp646 and Ser74, which acquire an exposure profile similar to the values in plasmin. The presented photonic mechanism of plasminogen activation has the potential to be used in clinical applications, possibly together with other enzymatic treatments for the elimination of blood clots.

## Introduction

Human blood plasma contains a large number of proteins and enzymes that regulates thrombosis (blood coagulation) and thrombolysis (dissolution of coagulated blood). The key enzyme in thrombolysis is plasmin, formed after activation of the inactive proenzyme plasminogen. Plasmin is a trypsin-like serine protease, which degrades fibrin. Fibrin is a protein that spontaneously polymerises to form blood clots, a mesh-like structure that covers a wound. Plasmin secures blood fluidity upon dissolution of fibrin thrombi (blood clots). Plasmin also plays a role in tissue remodelling (e.g. wound healing), angiogenesis, ovulation, embryo implantation onto the uterus, activation of some growth hormones and metalloproteinases [1].

Plasminogen activation in humans occurs by proteolysis and is predominantly catalysed by two serine proteases—the tissue-type Plasminogen Activator (tPA) or the urokinase-type Plasminogen Activator (uPA). The tPA has large affinity for fibrin and is the main activator in blood. Since uPA has affinity for a specific plasma membrane receptor, it is responsible for localised plasminogen activation in tissues and vessel walls [2]. Plasminogen can also be activated by a complex consisting of free plasminogen or plasmin molecules in tight association with streptokinase or by staphylokinase alone [3]. Plasminogen activation using streptokinase is not the natural activation mechanism in humans but along with tPA and uPA it is used in clinical therapy as thrombolytical agent for treatment of blood clotting disorders, e.g. myocardical infarction [4].

**Fig 1:**
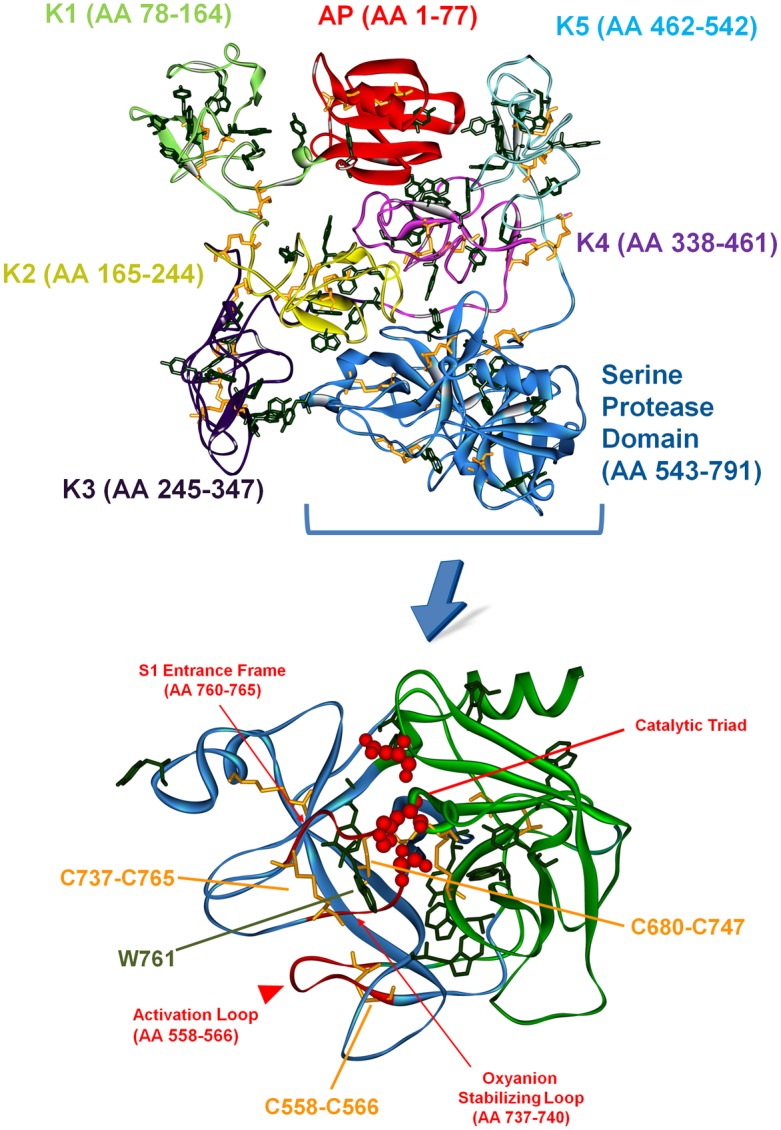
Tertiary structure of native human plasminogen. Top panel: Full length native 3D structure of native human Glu1-Pmg. The activation peptide (AP), the 5 kringle domains (K1 to K5), and the serine protease domain (where the catalytic site is located) are highlighted with different colors. The 24 disulphide bonds are displayed in orange in stick configuration, and the Trp and Tyr residues are displayed in dark green in stick configuration. Bottom panel: detailed 3D structure of the serine protease domain of human Glu1-Pmg (Ala543-Asn791). The C and N subdomains of plasminogen are displayed in blue and green, respectively. The disulphide bonds are displayed in stick configuration (orange) and two important disulphide bonds are highlighted: Cys556-Cys566 and Cys737-Cys765. The Trp and Tyr residues in the Serine Protease domain are displayed in line configuration (dark green). The active site residues (catalytic triad His603, Asp646 and Ser741 for plasminogen) are displayed as red CPK (0.5 Å radius). The three important loops involved in plasminogen activation are displayed in red: the oxyanion stabilizing loop, the S1 entrance frame and the activation loop. The xyz atomic coordinates for 3D representation were extracted from the crystallized structures of Glu1-Pmg (4A5T.pdb) [41].

The 3D structure of full-length native human plasminogen is displayed in Fig. 1 (top panel). It contains seven domains with a total of 24 disulphide bonds. The N-terminal activation peptide (AP) comprises residues Glu1-Lys77 (Fig. 1) and is stabilized by two disulphide bonds. It confers native plasminogen (also called Glu1-Pmg) its closed conformation [5]. Removal of this domain by proteolytic hydrolysis of Lys77-Lys78 peptide bond yields Lys78-Pmg [5, 6], characterized by a less compact structure and that is more effectively activated than Glu1-Pmg [5, 7]. Human plasminogen has five homologous triple-loop structures called kringles (K1 to K5, residues Lys78-Ala542, approximately 80 residues each, Fig. 1) [6]. Each of the kringle domains is stabilized by three intra-chain disulphide bridges. Furthermore, the connection between kringle 2 and 3 is reinforced by the presence of an additional inter-kringle disulphide bridge (Cys169-Cys297) [6]. The kringle domains contain lysine binding sites (LBS) that bind fibrin. When fibrin is not present, plasminogen adopts a closed and compact conformation. Upon binding to fibrin, plasminogen adopts an open conformation and is more easily activated [8]. The kringle domains interact with lysine-like ligands [5] and assist plasminogen in binding to large substrates (e.g. fibrin) [9], mammalian cells surfaces [10], bacterial proteins [11–13] and small ligands (e.g. Cl^-^, α, ω-amino acids [14, 15]). These interactions are also a part of the regulation mechanisms of plasminogen activation [5].

**Fig 2:**
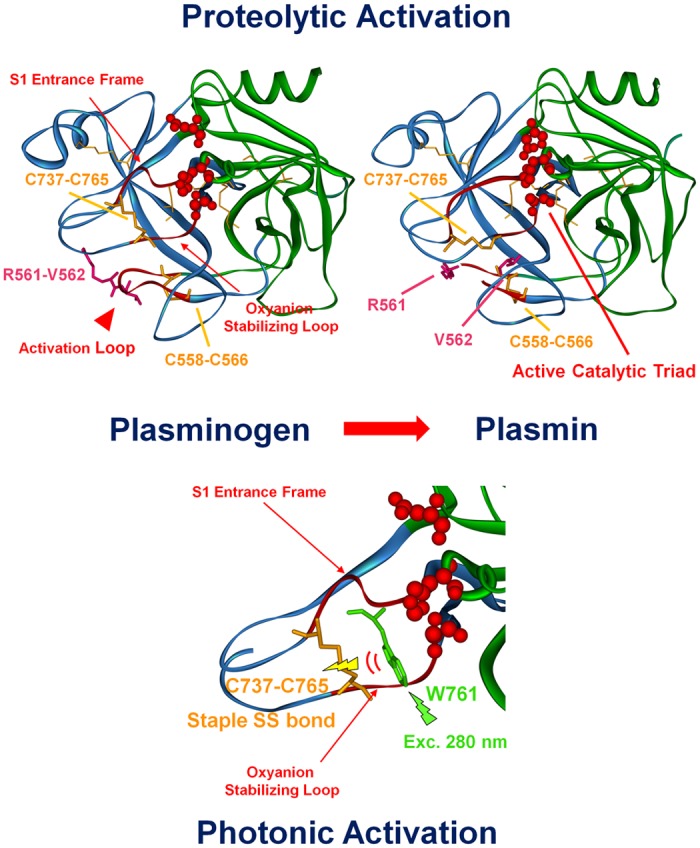
Proteolytic and photonic activation of plasminogen. 3D structures of the catalytic serine protease domain in plasminogen (to the left) and plasmin (to the right) (Ala543-Asn791). The xyz atomic coordinates for structural display were extracted from the crystallized structures of Glu1-Pmg (4A5T.pdb) [41] and activated plasminogen upon tight association with streptokinase (1BML.pdb) [4]. The C and N subdomains of plasminogen are displayed in blue and green, respectively. The disulphide bonds are displayed in stick configuration (orange) and two important disulphide bonds are highlighted: Cys556-Cys566 and Cys737-Cys765. The Trp and Tyr residues in the Serine Protease domain are displayed in line configuration (dark green). The active site residues (catalytic triad His603, Asp646 and Ser741 for plasminogen, His603, Asp646 and mutated Ala741 for plasmin) are displayed in CPK red (0.5 Å radius). The three important loops involved in plasminogen activation are displayed as red CPK: the oxyanion stabilizing loop, the S1 entrance frame and the activation loop. Top panel: The peptide bond Arg561-Val562 (in pink) in the activation loop is cleaved upon streptokinase activation leading to a conformational change and activating the catalytic triad (plasmin image to the right). Bottom panel: we can see in more detail the region around the oxyanion stabilizing loop, the S1 entrance frame. We observe a conformational change upon plasminogen activation involving the disulphide bond Cys737-Cys765 and Trp 761. We assume that upon UV illumination of plasminogen, Trp761 transfers its excitation energy to Cys737-Cys765. This results in disruption of this disulphide bond, leading to a conformational change that renders the catalytic triad active.

The inactive pro-enzyme domain of plasminogen is located at the C-terminus of the protein (Ala543-Asn791) [7] (Fig. 1, top and bottom panels). It is a typical serine protease catalytic domain, homologous to trypsin [16], belonging to the chymotrypsin family of serine proteases [7] and is commonly called microplasminogen [7]. The catalytic domain of human plasminogen is displayed in Fig. 1 (bottom panel). It contains four disulphide bonds and is has two subdomains, an N domain (displayed in green) and a C domain (displayed in blue). The active site residues His603, Asp646 and Ser741 (catalytic triad, CPK, in red) are located in a cleft at the junction of the two subdomains. Plasminogen catalytic activity is very reduced compared to plasmin, which is characterized by a 10^6^ times higher catalytic efficiency (*k_cat_*/*K_M_*) [8]. Conversion of the proenzyme plasminogen to plasmin renders the serine protease domain active. The proteolytic activation of plasminogen occurs upon cleavage of a specific peptide bond (Arg561—Val562), located in the so-called activation loop (see Fig. 2, top panel) [5, 6, 8]. Most of the structural modifications which occur upon plasminogen proteolytic activation occur in the C subdomain (Fig. 2, top panel), where the activation loop is (residues 558–566, in red) [8]. The activation loop is restrained by the disulphide bond Cys558-Cys566. Cleavage of the peptide bond Arg561-Val562 leads to a conformational change in two loops close to the catalytic triad: the oxyanion-stabilizing loop (residues 737–740, displayed as red ribbon) and the S1-entrance frame (residues 760–765, displayed as red ribbon) (see Fig. 2, top panel) [8]. The two loops are linked together by the disulphide bond Cys737-Cys765. Upon proteolytic activation, the location and conformation of the disulphide bond Cys737-Cys765 changes, while the location of Cys558-Cys566 does not (Fig. 2, top panel) [8]. Cys737–765 is characterized by a particular bond geometry, -RH Staple. Staple disulphide bonds have a high torsional potential energy, corresponding to regions of stress in a protein structure. Instead of acting as structure stabilizers, this type of disulphide bonds may have a functional redox role [17]. In fact, -RHStaple geometry has been associated to allosteric disulphide bonds in proteins [18]. These bonds are functional disulphide bonds and they control protein function by mediating conformational change when they undergo reduction or oxidation [18].

Usually, disulphide bonds are reduced chemically, enzymatically, or electrochemically. Reduction of disulphide bonds can also occur upon UV excitation. In proteins, it can occur directly upon cystine excitation at ~250 nm (wavelength of maximum absorption for dimethylsulfide, model for cystine absorption [19]), or indirectly upon UV excitation of the side chains of aromatic residues such as tryptophan (Trp, Abs^max^ ~278 nm [20]), tyrosine (Tyr, Abs^max^ ~275 nm [21]) and phenylalanine (Abs^max^ ~257 nm [20]). Reduction of disulphide bridges upon UV excitation of aromatic residues has been shown for proteins such as cutinase and lysozyme [22–24], bovine serum albumin [25, 26] prostate specific antigen [27], alpha-lactalbumin [28], antibody Fab fragments [29], and insulin [30]. Such mechanism is favoured by the spatial proximity between aromatic residues and disulphide bonds, which is a conserved structural feature in proteins [31]. Disulphide bond disruption upon UV excitation of aromatic residues can occur via electron transfer to cystines. Cystines can capture solvated electrons generated upon Trp and Tyr photoionization (scheme 1) or by direct electron transfer from ^3^Trp and ^3^Tyr triplet states formed upon UV excitation (scheme 2) [22, 32, 33]. Electron capture by cystines results in the formation of RSSR^.-^ (disulphide electron adduct) that can cleave to form a thiyl radical (RS.) and a thiol (RSH) (scheme 3) [22, 32]. Furthermore, solvated electrons can interact with the peptide chain generating hydroxide ions and ketyl radicals (scheme 4), which can propagate along the peptide chain [34, 35]. Entrapment of a ketyl radical by a disulfide bridge can yield a disulphide anion and lead to disulphide bridge breakage. Disulphide anion protonation can also lead to disulphide bond disruption (scheme 5) [32]. The thiyl radical RS. formed upon disulphide bond disruption can react with other amino acids, oxygen, or a second thiyl radical reforming a disulphide bond either within a protein or between proteins leading to aggregation [36].

$${\text{e}}_{\text{aq}}^{-}+\text{RSSR}\to \text{RSSR}{\;}^{\;•\;-}$$ (1)

T3yr+RSSR→Tyr•++RSSR•− (2)

$${\text{RSSR}}^{\;\;•\;-}\Leftrightarrow {\text{RS}}^{\;\;•}+{\text{RS}}^{\;-}$$ (3)

$${\text{e}}_{\text{aq}}^{-}+\;-\text{CONH}-\;\to \;{\text{OH}}^{\;-}+-\overset{•}{\text{C}}\left(\text{OH}\right)\text{NH}-\;$$ (4)

$${\text{RSSR}}^{\;\;•\;-}+{\text{H}}^{+}\Leftrightarrow \text{RS}{\;}^{\;•}+\text{RSH}$$ (5)

**Fig 3:**
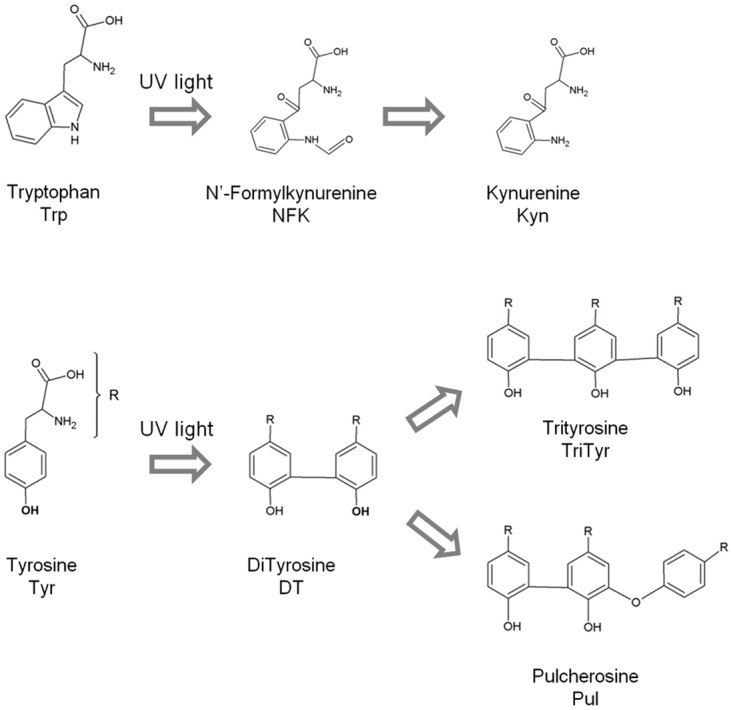
Chemical Structures of tryptophan, tyrosine and of the most important side-products formed upon their photo-oxidation.

Besides disulphide bond disruption, UV excitation of Trp and Tyr in proteins can trigger other photophysical and photochemical processes. It can lead to the formation of distinct Trp and Tyr side-products such as N-formylkyneurenine (NFK), kyneurenine (Kyn), dityrosine (DT) and isodityrosine, trityrosine and pulcherosine [36–38]. In Fig. 3 and Table 1 are summarized the major photoproducts generated upon Trp and Tyr photooxidation, their absorption and fluorescence spectral characteristics.

**Table 1:** Absorption and fluorescence spectral characteristics of tryptophan, tyrosine and the major side-products formed upon their photo-oxidation.

		**Absorption Maximum (Abs^max^) Extinction Coefficient (ε)**	**Excitation Maximum (Exc^max^)**	**Emission Maximum (Em^max^)**
Tryptophan (Trp)		278, 287, (272) nm ^a^ ε^278nm^ = 5579 M^-1^.cm^-1^ ε^287nm^ = 4954 M^-1^.cm^-1^ ε^272nm^ = 5360 M^-1^.cm^-1^	282, 290 nm ^b^	305–350 nm ^b^
N’-Formylkynurenine NFK	Ac-NFK-NH3 *Neutral*	261, 322 nm ^c^		420–434 nm ^c^
	NFK *Neutral/Acidic*	260, 321 nm ^d^	265, 330 nm ^e^	440 nm ^e^
	NFK *Alkaline*		240, 315 nm ^e^	400 nm ^e^
Kynurenine Kyn	Ac-Kyn-NH3	258, 360 nm ^c^		434–480 nm ^c^
Tyrosine Tyr	*Neutral/Acidic*	275 nm ^f^ ε^275nm^ = 1400 M^-1^.cm^-1^	275 nm ^b^	303 nm ^b^ ^,^ ^c^
	*Alkaline* (Tyrosinate)	290 nm ^f^ ε^290nm^ = 2300 M^-1^.cm^-1^		345 nm ^b^ ^,^ ^g^
Dityrosine DT	*Acidic*	284 nm ^h^ ε^284nm^ = 5400 M^-1^.cm^-1^	284 nm ^h^	409 nm ^h^
	*Alkaline*	316 nm ^h^ ε^316nm^ = 8600 M^-1^.cm^-1^	317 nm ^h^	407 nm ^h^
	*In proteins*			400–401 nm ^i^
Trityrosine TriTyr	*Acidic*	286 nm ^h^ ε^286nm^ = 11000 M^-1^.cm^-1^	286 nm ^h^	409 nm ^h^
	*Alkaline*	322 nm ^h^ ε^322nm^ = 11500 M^-1^.cm^-1^	319 nm ^h^	416 nm ^h^
Pulcherosine Pul	*Acidic*	282 nm ^h^ ε^282nm^ = 7700 M^-1^.cm^-1^	283 nm ^h^	416 nm ^h^
	*Alkaline*	315 nm ^h^ ε^315nm^ = 9500 M^-1^.cm^-1^	320 nm ^h^	414 nm ^h^

References:

^(a)^ [20];

^(b)^ [81];

^(c)^ [38];

^(d)^ [82];

^(e)^ [65];

^(f)^ [21];

^(g)^ [83];

^(h)^ [84];

^(i)^ [37].

Our study reports the consequences of 280nm excitation on the fluorescence properties, structure and biofunctionality of plasminogen. Continuous UV exposure of plasminogen (2.3 W.m^-2^) leads to disulphide bond breakage mediated by Trp and Tyr excitation, and to the formation of dityrosine and possibly NFK. Despite the onset of chemical modifications and structural changes, we observe that most of the protein fold is maintained after 10 min of 280 nm illumination. Circular dichroism confirms losses in secondary content of the protein, but not in the tertiary organization surrounding the protein aromatic residues. The overall fold of the protein is also retained after 10 min illumination (2.1W.m^-2^) since the melting temperature of the protein does not change after illumination. We show that 280 nm illumination of human plasminogen also leads to its activation into plasmin. After only 10 min of 280 nm illumination a 2.6 fold increase in proteolytic activity was observed. This increase in proteolytic activity is correlated with the likely UV light induced reduction of the allosteric disulphide bond C737-C765, with the effects of such reduction on the solvent accessibility of the catalytic triad and with the plasticity of the active site.

## Materials and Methods

### Protein and buffer preparation

Milli-Q water was used for buffer preparation (conductivity below 0.2 μS.cm^-1^).

Plasminogen from human plasma was purchased from Sigma-Aldrich (Sigma-Aldrich Danmark A/S, Copenhagen, Denmark) in powder form. In the majority of the experiments and unless stated differently we have used the Sigma-Aldrich plasminogen product P7999. The product was dissolved directly in 20 mM Lysine buffer pH 7.2 (Fluka, 62840) in order to make stock solutions and stored at ~-20°C in 50–100 µl aliquots until use. Prior to use the protein aliquots were slowly thawed at 4–8°C before dilution in the experimental buffer.

Plasminogen concentrations were determined by Abs^280nm^ using a molar extinction coefficient of 152200 M^-1^.cm^-1^, estimated using the bioinformatic tool ProtParam (Expasy, [39], entry: uniProt sequence P00747 [AA 20–810] for human plasminogen).

### Fluorescence Studies

UV illumination of plasminogen solutions was carried out in a RTC 2000 PTI spectrometer (Photon Technology International, Canada, Inc.347 Consortium Court London, Ontario, Canada) with a T-configuration, using a 75-W Xenon arc lamp coupled to a monochromator.

A 0.75 μM plasminogen work solution was prepared upon 36 X dilution of 27.3 µM plasminogen stock in 10 mM Tris HCl pH 8.0. Two mL of the work solution was placed in a quartz macro cuvette (1 cm path length) and continuously illuminated at 280 nm during 45 min. Emission and excitation spectra were recorded before and after illumination. Emission spectra were acquired with 280 nm, 295 nm, 325 nm, and 365 nm excitation. Excitation spectra were recorded with the emission fixed at 330 nm, 405 nm, 434 nm and 480 nm.

In order to collect the kinetics of fluorescence emission intensity at at 330 nm and 405 nm the experiment above was also carried out for different 280 nm illumination time periods. For each illumination session, a 0.55 μM plasminogen work solution was prepared upon 50 X dilution of 27.6 µM plasminogen stock onto 10 mM Tris HCl pH 8.0. Two mL of the work solution was placed in a quartz macro cuvette (1 cm path length) and continuously illuminated at 280 nm during 10 min, 20 min, 30 min, or 45 min. A fresh sample was used for each illumination session. Time-based fluorescent emission kinetic traces (emission fixed at 330 nm or 405 in the detector) were obtained during continuous 280 nm excitation. Before and after each illumination session, emission spectra were acquired upon 325 nm excitation.

Slits (bandpass) were set to 5 nm. Lamp power at 280 nm was 148 µW at the sample location. The illumination spot was 0.64 cm^2^. Irradiance was therefore 2.3 W.m^-2^.

In all experiments, samples were magnetically stirred at 950 rpm in order to secure homogeneous illumination. Solution temperature was set to 20°C using a Peltier element at the cuvette holder location.

### Detection of thiol groups’ concentration formed upon UV illumination of plasminogen

Plasminogen solutions were UV illuminated using the same experimental set-up, conditions and parameters as described in the previous section. Before each experiment, a plasminogen work solution (0.97 µM) was freshly prepared upon 40 X dilution of 38.7 µM plasminogen stock. Plasminogen samples (2 mL each) were illuminated at 280 nm in a quartz macro cuvette (1 cm path length) during 22.5 min, 45 min, 90 min, or 112.5 min.

Detection of free thiol groups was carried out using the Ellman’s assay [30, 40]. Ellman’s reagent, 5,5’-dithiobis-2-nitrobenzoic acid (DTNB) was purchased from Molecular Probes (product D8451, Life Technologies, Naerum, Denmark). One hundred mM stock solution was prepared in DMSO and stored at 4°C. After each illumination session, 1 mL of illuminated plasminogen solution was mixed with an excess of DTNB (10 µL of 100 mM stock solution). The molar ratio DTNB/plasminogen was ~1031. Four minutes after mixing the two components (sample kept in the dark), the absorbance intensity at 412 nm was measured in a UV/Visible spectrophotometer (UV1 VWR International—Thermo Electron Corporation, Thermo Fisher Scientific Inc. 81 Wyman Street Waltham, MA, USA), using a 1 cm path length quartz cuvette. Absorbance at 412 nm is due to the release of the product 2-nitro-5-thiobenzoate ion (TNB^2−^) and is proportional to the amount of thiol groups present in solution. The concentration of thiol groups was determined using an extinction molar coefficient for TNB^2−^ of 14150 M^−1^.cm^−1^ at 412 nm [40].

### Circular Dichroism measurements

The plasminogen stock solutions (110 µM) were prepared in 20 mM Lysine Buffer and this time stored at ~-80°C until use. Plasminogen samples (100 µl, 10 µM) were made up in 50 mM Tris buffer containing 100 mM NaCl, pH 7.4, and kept on ice all the time, except for illumination and optical measurements at 20ºC.

UV illumination was carried out in a ChronosBH spectrometer (ISS) with a T-configuration, using a 300-W Xenon arc lamp coupled to a monochromator. For illumination, plasminogen samples (100µl, 10 µM) was placed in an 10 × 2 × 5 mm (length×width×height) inner volume quartz cell with self-masking solid black walls and three clear quartz windows (Hellma) and continuously excited with 280 nm light for 10 min. The excitation beam was shaped to a rectangular area 2 × 10 mm (width x Height) and passed centrally through the excitation volume. Excitation slits were set to 4 nm. The irradiance was measured at 280 nm by an optical power meter (1917-R, Newport) placed in the excitation beam leaving the cell (2.1 W/m^2^). The temperature of the solution was kept at 20°C using a Peltier element at the cuvette holder location.

The CD measurements were carried out on a JASCO J-815 CD spectrometer (JASCO Corporation, Ishikawa-cho Hachioji-shi, Tokyo, Japan). A fresh non-illuminated plasminogen sample (100µl, 10µM) was prepared before the CD measurements. One hundred µL of 10 µM plasminogen solution (non illuminated or 10 min illuminated at 280 nm) was placed in a quartz microcuvette with a path length of 0.1 cm and a CD spectrum was acquired between 400 and 185 nm (comprising both near and far UV regions). The following parameters were set for the measurements: 1.0 nm band width, resolution 0.5 nm, 3 accumulations, scan speed 20 nm/min, sensitivity high, 16 s response time. The temperature was always kept at 10°C using a Peltier element at the cuvette holder’s location. The buffer signal was subtracted from all spectra.

**Circular dichroism based protein thermal unfolding studies**. The solution and protein preparation, and the experimental set-up, conditions and parameters used for UV illumination were the same as described in the previous section (Circular dichroism experiments).

The thermal unfolding of plasminogen samples (100µl, 10µM) was studied using CD spectroscopy by monitoring the intensity of the ellipticity signal of the protein during progressive heating at two different wavelengths (206 nm and 283 nm). The ellipticity intensity at 206 nm or 283 nm of a non illuminated plasminogen sample and of a UV illuminated plasminogen sample (10 min at 280 nm) was continuously monitored from 25ºC to 90ºC. The heating rate was 1ºC/min. A point was acquired every minute. Bandwidth was 1 nm and the response time was 16 s.

### UV Activation of Plasminogen

In this experiment, the plasminogen product used was Sigma-Aldricht P5661 (lyophilized plasminogen). As before, the powder was dissolved in 20 mM Lysine buffer pH 7.2 in order to make 18 µM (~1.7 mg ml^-1^) stock solutions and was stored at -20°C in 50 µL aliquots until use. A 50 µl aliquot was thawed slowly at 4–8°C before diluting 5x (to 3.6 µM) in 10 mM Tris HCl pH 8.0.

For each experiment, approximately 55 µL of the 3.6 µM plasminogen sample was added to an “illumination chamber” comprised by a 1 mm thick quartz slide, a rubber o-ring (6 mm in diameter and 1.5 mm thick), and a plexi-glass back block. The illumination chamber was placed in a dark box with the UV transparent quartz slide facing towards the exit slit opening of a PTI (Photon Technology Int.) monochromator connected to a 75 W Xenon Arc lamp source. The monochromator was set at 280 nm (+/- 6nm). The distance from the slit opening to the quartz slide of the illumination chamber was 2 cm. The sample was illuminated for 10 minutes at 25°C (+/- 0.5°C). As a negative control, the same procedure (with approximately 55 µl sample in the illumination chamber) was carried out in the absence of UV illumination.

### Plasmin Activity Measurements

A standard fluorescent substrate for testing the activity of a wide range of proteases (Molecular Probes, Enzcheck E-6639) was used. The substrate (casein based and labelled with Bodipy dye) was diluted according to the suppliers’ instructions in 10 mM Tris HCl at pH 8.0.

45 µL of sample (illuminated or non-illuminated, preparation described in the section above) was mixed with 55 µL substrate solution in a black 96-well microtiter plate and incubated in darkness at ambient room temperature (20–23°C). The samples were analysed in a SpectraMax XS Fluorescence Reader (excitation at 590 nm / emission at 640 nm with cut-off at 610 nm). The fluorescence emission intensity emitted by the fluorescent cleaved products was measured after 1 h and 22 h of incubation.

### Molecular Dynamics Simulations

To investigate the structural role of the disulphide bond Cys737-Cys765, molecular dynamics simulations of the catalytic domain (542–791) of native plasminogen, plasminogen reduced in that bond, and plasmin were performed. The x-ray structures with pdb code 4A5T [41] and 1BML [4] were used to initiate the simulations of the plasminogen forms and plasmin, respectively. Three simulations of 30 ns were performed for each protein in water using the simulation package GROMACS 4.5.5 [42], the force field GROMOS 54A7 [43], and the water model SPC [44]. The simulation boxes were filled with 12997–13724 water molecules and the system was neutral, with the counter-ions Na^+^ and Cl^−^ in a concentration of 10 mM. The non-bonded interactions were treated using a twin-range cutoff of 9/14 Å for the van der Waals, a neighbor lists update every 10 fs, and the particle mesh Ewald method [45] to treat long-range electrostatics beyond a 9 Å cutoff. Protein and solvent were separately coupled to temperature baths at 298 K using the velocity-rescaling thermostat [46] (temperature coupling of 0.1 ps) and pressure was kept at 1 atm using the Berendsen coupling [47] (isothermal compressibility of 4.5 × 10^−5^ bar^−1^ and pressure coupling of 0.5 ps). The time step was 2 fs, and all bonds were constrained with the LINCS algorithm [48]. A standard protocol was used to minimize and relax the system before running the simulations. Monitoring of various properties indicated that all simulations were essentially equilibrated after the first 15 ns, which were thus discarded prior to analysis.

### Data Analysis

**Structure Analysis**. The crystallography data used for the display of the 3D protein structure (Figs. 1 and 2) and distance calculations was extracted from the PDB files 4A5T.pdb (full-length native human plasminogen, 3.49 Å resolution [41]) and 1BML.pdb (human plasmin catalytic domain in complex with streptokinase, 2.90 Å resolution [4]). Distances between Trp, Tyr residues and disulphide bonds, and among Tyr residues in native human plasminogen were calculated in Matlab R20120B after extracting the atomic xyz coordinates from the PDB file 4A5T.pdb. For the calculation of distances between aromatic residues (Trp and Tyr) and disulphide bonds we have considered the shortest distances between atoms of each pair of elements (Trp, Tyr and disulphide bonds). We have considered all the atoms in the disulphide bonds. In the case of Trp and Tyr, only the atoms belonging to the indole and benzene rings were considered. For the calculation of distances between Tyr residues, we have considered the shortest distances between the carbon atoms in the ortho-position(s) of the phenol group.

**Fig 4:**
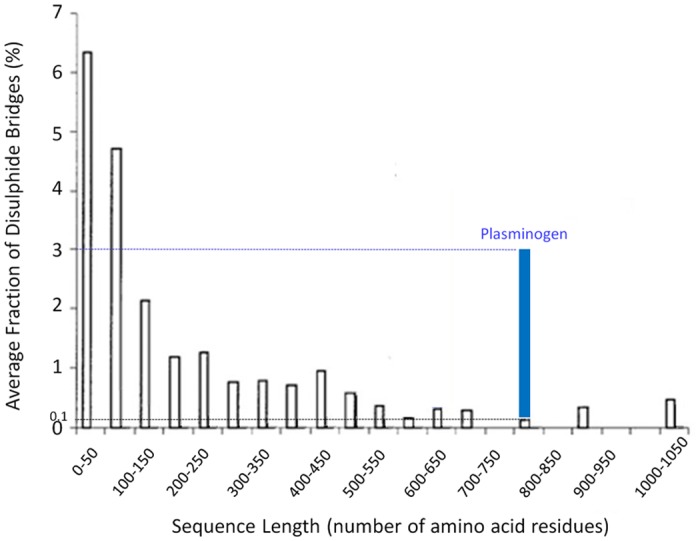
Dependence of the average fraction of disulphide bridges on protein chain length. The fraction of disulphide bridges was obtained by dividing the number of disulphide bridges found in a protein by the protein sequence length (number of amino acids) multiplied by 100. The fraction of disulphide bridges estimated for human plasminogen (in blue, 3.1%, for 767 amino acids) is much higher than what would be expected for a protein of about that length (0.1% for 700–750 amino acids).

The fraction of disulphide bridges in human plasminogen and other proteins was obtained upon dividing the number of disulphide bridges found in a protein by the protein chain length (number of amino acids) and multiplying by 100 (Fig. 4). The PDB dataset used for the calculation on Fig. 4 (dependence of the average fraction of disulphide bridges on the protein chain length) has been published previously by our group [31].

The disulphide bonds of human plasminogen has been also analysed using the software tool *Disulphide Bond Analysis* available online from the webpage of the Adult Cancer Programme (University of New South Wales, Australia, [49]). The software provides different geometric measures, secondary structural information (including disulphide bond configuration) and solvent-accessibility values for the disulfide bonds of a protein upon uploading its xyz atomic coordinates. The PDB file 4A5T.pdb (full length native human plasminogen, 3.49 Å resolution [41]) was submitted to the online software for analysing the disulphide bonds in human plasminogen. The value considered for the total solvent accessible surface area (ASA) of a Cys residue is 104 Å (Cys side chain). This value was estimated considering Cys in the tripeptide Gly-Cys-Gly with the main chain in an extended conformation [50].

**Emission Spectra (280 nm, 295 nm, 325 nm, and 365 nm excitation)**. Emission spectra were corrected by subtracting the spectra recorded for the buffer solution. Subsequently the spectra were smoothed using 5 points adjacent averaging, except for the emission spectra recorded upon 365 nm excitation, which were smoothed using a 7 points adjacent averaging. Normalized emission spectra were obtained by dividing each data point by the maximum intensity value in each spectrum.

**Fig 5:**
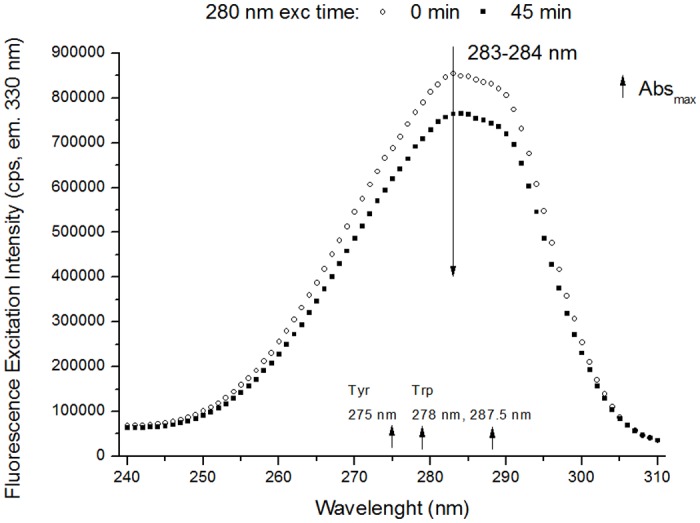
Fluorescence excitation spectra of human plasminogen (330 nm emission). The excitation spectra (em. 330 nm) were obtained before and after 280 nm light continuous illumination (45 min) of human plasminogen in solution. After 45 min of 280 nm illumination there is a decrease in excitation intensity at ~283 nm. Small increasing arrows indicate the wavelengths of maximum absorption (Abs_max_) of Trp and Tyr in solution.

**Excitation Spectra (emission fixed at 330 nm, 405 nm, 434 nm and 480 nm)**. The excitation spectra were first corrected for buffer contribution (e.g. data displayed in Fig. 5). The spectra with emission fixed at 405 nm, 434 nm and 480 nm were smoothed using adjacent averaging with 5, 7 and 9 points, respectively. Normalized excitation spectra were obtained by dividing each data point by the maximum intensity value in each spectrum.

**Fig 6:**
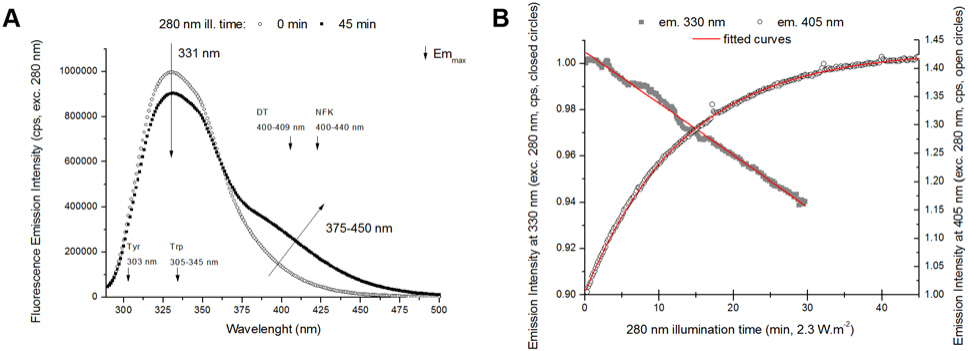
Fluorescence emission of human plasminogen (280 nm excitation). **A**- Fluorescence emission spectra (280 nm exc.) obtained before and after 280 nm light continuous illumination (45 min) of human plasminogen in solution. 280 nm illumination leads both to a decrease in emission intensity at 330 nm and to an increase in emission intensity between 375 nm and 450 nm. Small decreasing arrows indicate the wavelengths of maximum emission intensity (Em_max_) of Trp, Tyr, DT and NFK in solution. **B**- Fluorescence emission intensity kinetic traces obtained at 330 nm and 405 nm (exc. 280 nm) upon continuous illumination of human plasminogen with 280 nm light. Fitting of the experimental trace obtained with emission fixed at 330 nm and 405 nm was carried out using an exponential function *F(t)* = *C*1– *C*2.e^-*kt*^. The fitted parameter values and corresponding errors, and root mean square error values were obtained after fitting the kinetic trace (Table 5 and Materials and Methods).

**Time-based fluorescence emission kinetic traces (emission at 405 nm and 330 nm)**. The kinetic traces of fluorescence emission at 405 nm and 330 nm and excitation fixed at 280 nm (Fig. 6B) were obtained directly during continuous 280 nm illumination of plasminogen (1 point acquired each 10 s; 90 min illumination time for 405 nm em.; 30 min 280 nm illumination time for 330 nm em.). The traces were normalized by dividing each data point by the emission intensity value at 0 min illumination time.

**Fig 7:**
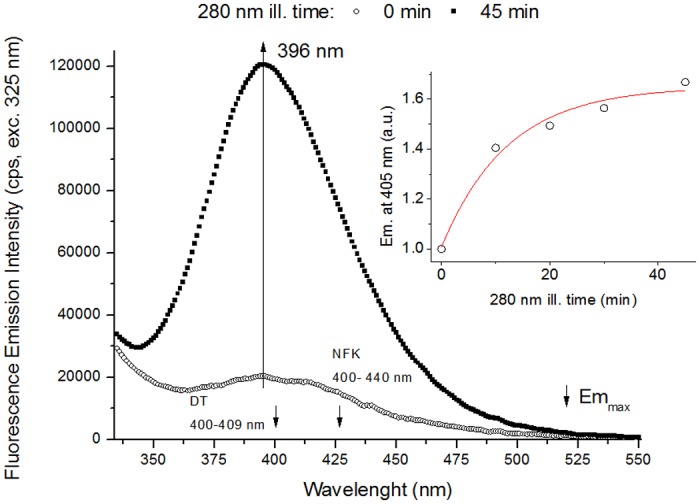
Fluorescence emission of human plasminogen (325 nm excitation). Fluorescence emission spectra (325 nm exc.) recorded before and after 280 nm light continuous illumination (45 min) of human plasminogen in solution. There is an increase in emission intensity at 396 nm with 280 nm ill time. Small decreasing arrows indicate the wavelengths of maximum emission intensity (Em_max_) of DT and NFK in solution. **Insert**- Fluorescence emission intensity at 405 nm (exc. 325 nm) *vs* 280 nm illumination time. The experimental points were fitted to an exponential function *F(t)* = *C*1– *C*2.e^-*kt*^. Fitted parameter values and corresponding errors, and root mean square error values were obtained after fitting the kinetic trace (Table 5).

The kinetic trace of fluorescence emission at 405 nm upon excitation at 325 nm displayed in Fig. 7 (insert) was obtained in a different way. The emission intensity values at 405 nm were obtained from the emission spectra recorded upon 325 nm excitation after 280nm illumination of the sample for different periods of time (10 min, 20 min, 30 min, 45 min, or 90 min).

**Circular dichroism far and near UV spectra**. The near UV CD part of the CD spectra (251–450 nm) was first corrected by baseline substraction. The baselines were created by interpolation (12 points for 0 min 280 nm ill. and 16 points for 10 min 280 nm ill.) using the Origin 8.0 tool peak analyser.

The full CD spectra data set (185–450 nm) was converted from the ellipticity machine units (milidegrees, mdeg) to mean residue ellipticity units [θ] (deg.cm^2^.dmol^-1^) using the formula (pathlength in mm and concentration in mg.mL^-1^):

$$\left[\theta \right]=\frac{\left(m\deg\times mean\_residue\_weight\right)}{\left(pathlength\times concentration\right)}$$

The mean residue weight of a protein is obtained by dividing the molecular weight by the number of backbone amides (number of amino acids—1 if the protein is not acetylated) [51].

The far UV CD spectra were analyzed using the online CD deconvolution tool Dichroweb (http://dichroweb.cryst.bbk.ac.uk/html/home.shtml, [52, 53]) in order to estimate the secondary structure of the protein. The deconvolution results were obtained using the algorithm CDSSTR [54–56] and the reference data set 4 [57], which provided the best fit for the experimental data.

**Fitting procedures**. The kinetic traces of fluorescence emission at 330 nm (280 nm exc., Fig. 6B) and 405 nm (280 nm exc., Fig. 6B, and 325 nm exc., Fig. 7 insert) were fitted with an exponential function F(t) = C_1_– C_2_.e^-kt^ for the window 0–30 min and 0–45 min, respectively. F(t) is the fluorescence emission intensity at 330 nm or 405 nm (a.u.) at 280 nm illumination time t (min), C1 and C2 are constants and k is the rate constant of fluorescence emission intensity decrease or increase (min^-1^). The fitted parameter values and corresponding errors, and root mean square error values obtained after fitting the 405 nm emission kinetic trace are presented in the Results section. For the kinetic trace of fluorescence emission at 330 nm the fitted parameters were as follows. The root mean square error R^2^ was 0.99262. The values recovered from the fitting for C1 and C2 were 7.37 ± 31.03 and 6.37 ± 31.03, respectively. The rate constants of fluorescence emission intensity decrease k1fitted value was -3.49E-04 ± 16.9E-04 min^-1^.

The kinetics of thiol group formation versus 280 nm illumination time have also fitted according to an exponential function *y = y_0_-A.e^-R0t^*, where *y* is the concentration of thiol groups (µM) at the 280 nm illumination time t(h), *y_0_* and *A* are constants and *R0* is the rate of thiol group formation (min^-1^).

The circular dichroism thermal unfolding curves (ellipticity at 206 nm and 283 nm) were fitted using a modified Boltzmann function (see Results section). For the thermal unfolding curve obtained with ellipticity fixed at 206 nm, the fitting was done for the interval 60–90°C. For the curve obtained with ellipticity fixed at 206 nm, the fitting was done for the data points 58.11–80.8°C and 57.35–80.78°C for non-illuminated and illuminated (10 min 280 nm) plasminogen samples, respectively. The fitting parameter x0 corresponds to the inflection point of the Boltzmann curve and the corresponding temperature is the melting point determined by circular dichroism.

Smoothing procedures, data fitting and plotting were done in Origin Pro 8.0.

## Results

### Bioinformatics

In Fig. 1 (top panel) is presented the 3D crystal structure of native human plasminogen. The five kringle domains of the protein are visible (K1 to K5), as well as the activation peptide (AP) and the catalytic serine protease domain. Human plasminogen contains 19 Tyr, and 30 Trp and 24 disulphide bonds, which are distributed over all domains of the protein.

**Aromatic Residues**. A large fraction of the aromatic residues is located in close spatial proximity of disulphide bonds. In human plasminogen, 14 of the 19 Trp residues, and 26 of the 30 Tyr residues are situated less than 8 Å away from an disulphide bond (less than the average size of an amino acid, which is ~ 10 Å [58]. In Table 2 are listed the Trp, Tyr-disulphide bond pairs whose inter-distance is less than 6 Å (threshold distance defined considering direct Van der Waals contact distance ≤5.2 Å [59]. We can find Trp, Tyr-disulphide bond pairs in or close to Van der Waals contact in all protein domains. In Fig. 1 (bottom panel) is displayed the catalytic domain of plasminogen, which contains 6 disulphide bonds, 6 Trp and 5 Tyr residues. In Table 3 are summarized the distances between Trp, Tyr residues and disulphide bonds in the serine protease domain (distance cut-off of 12 Å). Trp761 is the only Trp/Tyr residue in van der Waals contact (4.24 Å) to a SS bond, Cys737-Cys765 (Fig. 1, bottom panel). The latter links the oxyanion stabilizing loop (residues 737–740) and the S1 entrance-frame (residues 760–765). Tyr774 is also quite close to Cys737-Cys765 (8.35 Å). The other disulphide important for plasminogen activation, Cys558-Cys566 (Fig. 1, bottom panel), which restrains the activation loop, has only one Trp/Tyr in close spatial proximity (Trp573, 9.06 Å away).

**Table 2:** Shortest spatial distances between disulphide bonds and aromatic residues (tryptophan and tyrosine) in full-length native human plasminogen.

**Protein Domain**	**Disulphide Bond**	**Aromatic Residue**	**Distance (Å)**
**AP**	C34-C42	Y6	4.48
	C30-C54	Y47	4.18
**K1**	C133-C145	W108	5.83
		W144	5.93
**K2**	C215-C238	Y174	5.87
**K3**	C256-C333	Y254	4.13
	C277-C316	W280	5.74
**K4**	C407-C430	Y366	5.60
**K5**	C483-C524	W486	5.76
	C512-C536	Y470	5.87
**Serine Protease Domain**	C737-C765	W761	4.23

The shortest distances (< 6 Å) between atoms of each pair of elements (Trp, Tyr and disulphide bonds) were considered. For Trp and Tyr residues, only the atoms belonging to the indole and benzene rings were considered.

**Table 3:** Shortest spatial distances between disulphide bonds and aromatic residues (tryptophan and tyrosine) in the serine protease domain of human plasminogen.

**Disulphide Bond**	**Aromatic Residue**	**Distance (Å)**
C548-C666	W573	11.54
	W575	7.05
	Y672	11.97
	Y753	8.12
**C558-C566**	**W573**	**9.06**
C588-C604	W685	9.92
	W761	7.18
	Y614	7.30
C680-C747*	W573	9.44
	W575	10.25
	Y672	7.48
	Y753	7.25
C710-C726	Y774	7.06
C737–C765*	**W761**	**4.24**
	**Y774**	**8.35**

The shortest distances (<12 Å) between atoms of each pair of elements (Trp, Tyr and disulphide bonds) were considered. For Trp and Tyr residues, only the atoms belonging to the indole and benzene rings were considered.

* -RH Staple disulphide bonds.

In bold: disulphide bonds relevant for plasminogen activation.

In human plasminogen, one third of the Tyr residues are less than 6 Å (threshold distance defined considering direct Van der Waals contact distance ≤5.2 Å [59] away from another Tyr residue (data not shown). Three clusters of Tyr residues can be identified, where the closest distance between single Tyr residues is less than 6 Å: Tyr359-Tyr366, Tyr146-Tyr154-Tyr156, and Tyr429-Tyr535-Tyr533-Tyr525 (data not shown). In Table 4 are displayed the shortest distances between the carbon atoms involved in possible dityrosine cross-linking (ortho position(s) of the phenol group) for these 3 clusters.

**Table 4:** Shortest spatial distances between tyrosine residues in full-length native human plasminogen.

**Tyr1**	**Tyr2**	**Distance (Å)**
	**Cluster 1**	
Tyr359	Tyr366	7.64
	**Cluster 2**	
Tyr146	Tyr154	6.32
Tyr154	Tyr156	3.86
	**Cluster 3**	
Tyr429	Tyr535	4.41
Tyr525	Tyr533	6.00
Tyr533	Tyr535	5.94

The shortest distances between the carbon atoms in the ortho-position(s) of the phenol were considered.

**Disulphide Bonds**. The characteristics of the disulphide bonds of native human plasminogen are summarized in S1 Table. Two of the disulphide bonds of plasminogen have a –RHStaple geometry, Cys680-Cys747 and as already mentioned Cys737-Cys765, which is relevant for plasminogen activation. The disulphide bonds are ordered by increasing solvent-accessible surface area (ASA) of the Cys residues. Most of the disulphide bonds of plasminogen comprise cysteines with low ASA values. Out of the 24 disulphides, 15 of them have cysteine residues with ASA values lower than 7 Å, which corresponds to ~12% of the total ASA of a Cys residue. Only 7 of the disulphide bonds comprise Cys residues with ASA values superior to 32 Å, corresponding to 31% of the total ASA of a Cys residue. Among these disulphide bonds are Cys558-Cys566 and Cys737-Cys765, both relevant in plasminogen enzymatic activation. Cys737-Cys765 displays the second highest Cys ASA value, with 57 Å for Cys737 and 21 Å for Cys 765, corresponding to ~55% and ~20% of the total ASA of a Cys residue.

The fraction of disulphide bonds in plasminogen is displayed in Fig. 4 together with the dependence of the average fraction of disulphide bridges on the protein chain length [31]. Human plasminogen contains 767 amino acids. For a protein of that chain length size (about 700–750 amino acids) we would expect an average fraction of disulphide bridges of ~0.1%. However, the fraction of disulphide bridges obtained for human plasminogen is ~3%, indicating that this protein exceptionally rich in disulphide bridges.

### Steady State Fluorescence

Emission and excitation spectra were obtained prior and after illuminating human plasminogen with 280 nm (45 min, irradiance of 2.3 W.m^-2^) in order to study the effects of continuous UV illumination on the fluorescence properties of the protein.

**Excitation Spectra (emission 330 nm)**. In Fig. 5 is displayed the excitation spectra of plasminogen with fluorescence emission fixed at 330 nm, before and after continuous 280 nm illumination of the protein for 45 min. Both Trp and Tyr absorption contribute to this spectrum. Continuous 280 nm illumination leads to a decrease in the fluorescence excitation intensity. After 45 min of illumination, the excitation intensity at 283 nm decreases by 10.6%. The correspondent normalized excitation spectra (data not shown) show no shift in the wavelength where maximum excitation intensity is observed (~283 nm).

**Emission Spectra (excitation 280 nm)**. The emission spectra of plasminogen recorded upon 280 nm illumination before and after 45 min of continuous 280 nm illumination are shown in Fig. 6A. A decrease in the intensity of the fluorescence emission at ~330nm is observed after continuous UV illumination of the protein. Trp and Tyr residues in the protein contribute to the fluorescence emission observed from 305 to 345 nm. After 280 nm prolonged illumination, there is no wavelength shift of the most intense peak centered at ~331 nm. The emission intensity of plasminogen at 330 nm decreases by 9.4% after 45 min of continuous 280 nm illumination. An increase of the fluorescence emission intensity at 375–450 nm is observed after continuous UV illumination at 280 nm (Fig. 6A): fluorescence emission intensity increases by 144.3% at 405 nm and 330.8% at 434 nm.

In Fig. 6B are displayed the kinetics of fluorescence emission intensity at 330 nm and 405 nm upon continuous 280 nm illumination. There is a progressive decrease of the Trp and Tyr fluorescence emission intensity (at 330 nm) with increasing 280 nm illumination, which is correlated with a progressive increase of fluorescence emission intensity at 405 nm. Fitting the experimental data (in red) reveals that both kinetics are exponential. The fit results are presented in Materials and Methods and Table 5. After 10 min of 280 nm illumination the fluorescence emission intensity decreases by 1.4% at 330 nm and increases by 23.3% at 405 nm. After 45 min, the increase in emission intensity at 405 nm (exc. 280 nm) is up to 41.9%. This increase is lower than the calculated for the emission spectra (144.3%, Fig. 6A). The reason for this is that the emission spectra were corrected for the buffer contribution (Fig. 6A) and the kinetics were not, as it is not possible to correct the time-dependent 405 nm emission intensity values for the buffer contribution. Therefore, the kinetic traces take into account the initial 405nm fluorescence from the buffer that is not present in the corrected emission spectra (Fig. 6A). The initial 405 nm buffer fluorescence is due to the buffer component L-Lysine that fluoresces at 405 nm [60]. L-lysine fluorescence does not change along the 45 min of 280 nm illumination (data not shown).

**Table 5:** Parameter values recovered upon fitting the fluorescence emission kinetic traces recorded with fixed emission at 405 nm (exc. 280, Fig. 6B; or exc. 325 nm, Fig. 7 insert).

**Fitting Equation**		***F(t)* = *C*1− *C*2.e^-*kt*^**	
		**exc. 280 nm**	**exc. 325 nm**
**Fitting Parameters**	R^2^	0.99936	0.96489
	*C*1	1.43 ± 5.26E-4	1.65 ± 0.05
	*C*2	-0.43 ± 6.68E-4	-0.64 ± 0.07
	*k* (min^-1^)	0.076 ± 3.36E-4	0.082 ± 0.022

**Emission Spectra (excitation 325 nm)**. In order to verify the formation of fluorescent photoproducts of Tyr and Trp such as DT (absorbance maximum, Abs^max^ at 316 nm, see Table 1) and NFK (Abs^max^ at 321 nm, see Table 1), emission spectra were obtained upon 325 nm excitation prior and after 45 min of 280 nm illumination (Fig. 7). There is an increase in fluorescence emission intensity at 390–400 nm (exc. 325 nm) with continuous 280 nm illumination time. The fluorescence emission maximum is centred at ~396 nm, corresponding to the emission maximum of dityrosine (Em^max^ at 400–409 nm, see Table 1) and to a spectral region where NFK can also emit (for NFK, Em^max^ at 400–440 nm, see Table 1). The wavelength of maximum emission does not change with increasing 280 nm illumination time. After 45 min of continuous 280 nm illumination, the fluorescence emission intensity at 405 nm increases by 508.2% (Fig. 7).

The fluorescence emission intensity increase at 405 nm (325 nm exc.) with continuous 280 nm illumination follows a single exponential kinetics as can be observed in the insert of Fig. 7. The 405 nm fluorescence emission increases by 40.5 and 66.8% after 10 min and 45 min of illumination, respectively. The kinetics show a lower increase in emission intensity after 45 min than the obtained from the emission spectra (508.2%, Fig. 7). As explained in the previous section this occurs since the emission spectra are corrected for the buffer contribution and the kinetics are not.

In Table 5 are summarized the fitting parameters obtained upon fitting the kinetic traces of fluorescence emission increase at 405 nm (280 nm exc., Fig. 6B; 325 nm exc., Fig. 7 insert). The value obtained for the rate constant of fluorescence emission intensity increase *k* is similar for both excitation wavelengths (0.076 min^-1^ for 280 nm exc. and 0.082 min^-1^ for 325 nm exc.).

**Fig 8:**
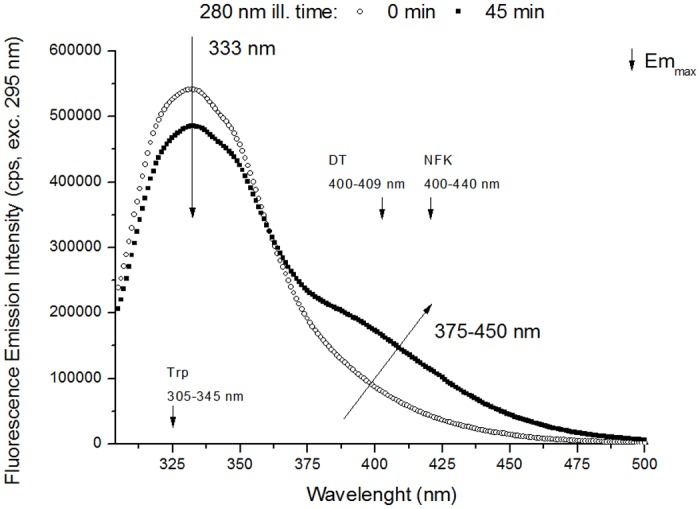
Fluorescence emission spectra of human plasminogen (295 nm excitation). Fluorescence emission spectra (295 nm exc.) recorded before and after 280 nm continuous illumination (45 min) of human plasminogen in solution. UV illumination of plasminogen results in a decrease in emission intensity at 330 nm and an increase in emission intensity between 375 nm and 450 nm. Small decreasing arrows indicate the wavelengths of maximum emission intensity (Em_max_) of Trp, DT and NFK in solution.

**Emission Spectra (excitation 295 nm)**. Fig. 8 shows the effect of 280 nm continuous illumination (45 min) on the emission spectra of plasminogen obtained upon 295 nm excitation. Among the aromatic residues, only Trp is excited at 295 nm. After 45 min of 280 nm illumination, there is a 10.6% decrease in fluorescence emission intensity at 330 nm. Normalization of the spectra (data not shown) shows that wavelength of maximum emission is 332 nm at illumination time 0 h and does not change with continuous UV illumination. After 45 min, the fluorescence emission intensity increases by 115.5% at 405 nm and by 192.5% at 434 nm.

**Fig 9:**
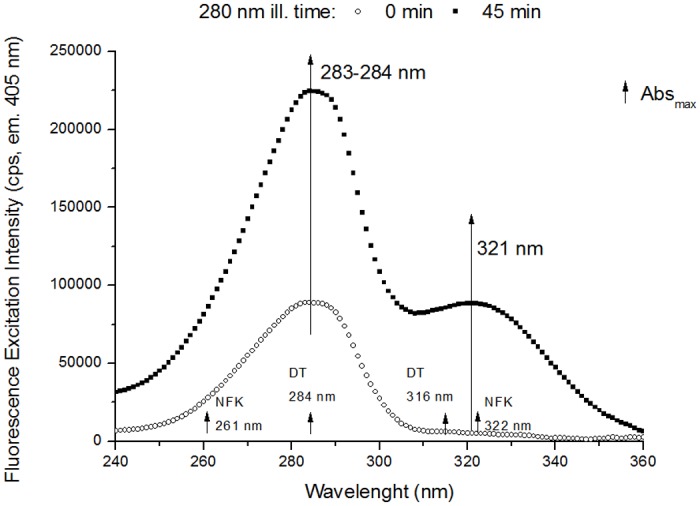
Fluorescence excitation spectra of human plasminogen (405 nm emission). Fluorescence excitation spectra (emission at 405 nm) obtained before and after 280 nm continuous illumination (45 min) of human plasminogen in solution. There is an increase in excitation intensity at the peak located at ~283–284 nm and a new peak is formed at 321 nm upon 280 nm illumination. Small increasing arrows indicate the wavelengths of maximum absorption (Abs_max_) of DT, Kyn and NFK in solution.

**Excitation Spectra (emission 405 nm)**. Excitation spectra (emission fixed at 405 nm) were obtained before and after continuous 280 nm illumination (45 min) in order to investigate which fluorescent species contributed to the increase in fluorescence emission intensity at 405 nm (Fig. 9). Before 280 nm illumination (0 min illumination), we can observe a single excitation peak centered at ~283 nm. Continuous 280 nm illumination leads to an increase in excitation intensity at 283 nm, and to the formation of a new peak, centred at ~321 nm. Both DT (Abs^max^ at 316 nm, see Table 1) and NFK (Abs^max^ at 321 nm, see Table 1) absorb at these wavelengths. After 45 min of 280 nm illumination, fluorescence excitation intensity increases by 151.4% and 1706.9% at 283 nm and 325 nm, respectively.

**Fig 10:**
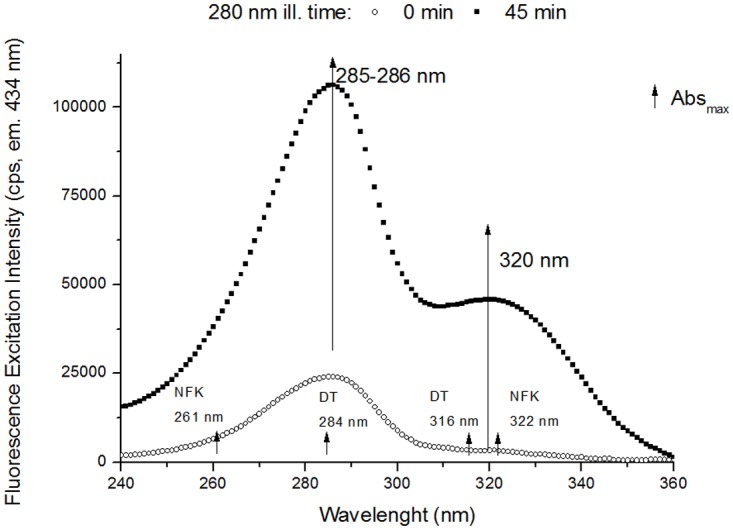
Fluorescence excitation spectra of human plasminogen (434 nm emission). Fluorescence excitation spectra (emission at 434 nm) obtained before and after 280 nm continuous illumination (45 min) of human plasminogen in solution. 280 nm illumination leads to an increase in excitation intensity at the peak located at ~285–286 nm and to the formation of a new peak at 320 nm. Small increasing arrows indicate the wavelengths of maximum absorption (Abs_max_) of DT, Kyn and NFK in solution.

**Excitation Spectra (emission 434 nm)**. In Fig. 10 are displayed the excitation spectra (emission fixed at 434 nm) obtained before and after continuous 280 nm illumination (45 min). The spectra were recorded in order to verify the presence of NFK (Em^max^ at 434 nm in water and neutral pH, [38], see Table 1). At illumination time 0 min, an excitation peak can be observed with maximum centred at ~285 nm. After 45 min of illumination, we can observe both an increase in excitation intensity at 285 nm and the presence of a new peak, centred at ~320 nm. After 45 min, the excitation intensity at 283 nm (where DT absorbs, Abs^max^ at 284 nm, see Table 1, dityrosine still emits at 434 nm) increased 4.4 fold relative to illumination time 0 min. At 327 nm, where DT (Abs^max^ at 316 nm, see Table 1) and NFK (Abs^max^ at 321 nm, see Table 1) absorb, there is a 15.4 fold increase in excitation intensity.

### Circular Dichroism measurements

Circular dichroism (CD) measurements were carried out in order to infer the effects of brief 280 nm illumination (10 min, irradiance of 2.1 W.m^-2^) on the secondary and tertiary structure of plasminogen.

**Fig 11:**
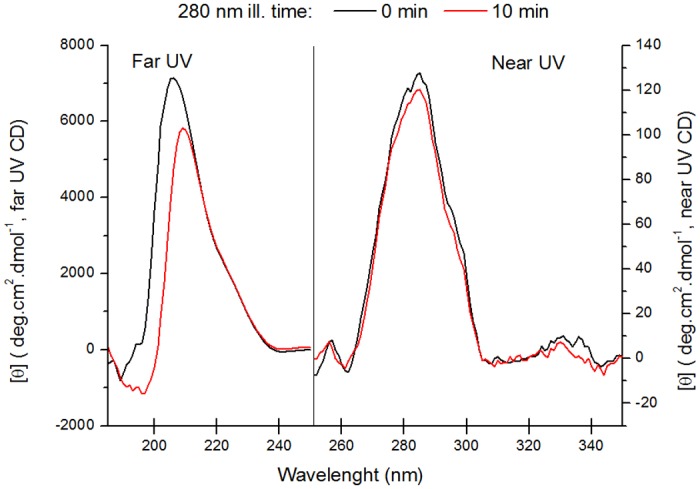
Far and near UV circular dichroism spectra of human plasminogen. Far UV (185–250 nm) and near UV (250–350 nm) CD spectra were obtained before and after 280 nm light continuous illumination (10 min) of human plasminogen in solution. 280 nm illumination leads to a loss of far UV ellipticity signal at 206 nm (33.4%) and to a red-shift in wavelength of maximum ellipticity (206 nm to 209 nm). There are almost no changes in the near UV CD spectrum of plasminogen with 280 nm illumination. After 10 min of 280 nm ill. the ellipticity signal at the wavelength of maximum ellipticity (285 nm) decreases only by 6%.

The far and near UV CD spectra of fresh (0 min 280 nm ill.) and UV illuminated plasminogen (10 min 280 nm ill.) are displayed in Fig. 11. Continuous 280 nm illumination of the protein leads to spectral losses in the far UV region of the CD spectrum (185–250 nm, Fig. 11). The far UV CD spectrum of fresh human plasminogen (280 nm ill. time 0 min) shows a single distinct positive peak at ~206 nm. 10 min of 280 nm illumination leads to a 3 nm red-shift of the peak to ~209 nm. The red-shift is correlated with a decrease in ellipticity signal with UV-illumination. After 10 min of 280 nm illumination, the ellipticity signal at 206 nm decreases by 33.4%. The secondary structural contents of both non illuminated and UV illuminated plasminogen were estimated upon deconvolution of the far-UV CD spectra (Table 6). The results show that the secondary structure of non illuminated human plasminogen is mostly constituted by β strand (76%) and turns (17%), while there is almost no presence of α helix (below 1%). UV-illumination (10 min 280 nm) leads to loss of β strand features (down to 31%), and a major increase in unordered structure (3% to 37%). The turn content also increases to 31% with UV-illumination.

**Table 6:** Quantification of the secondary structure of human plasminogen samples (non-illuminated, 0 min; UV illuminated, 10 min at 280 nm) obtained upon far UV CD spectra deconvolution with the algorithm CDSTRR.

**Time**	**Fraction of Secondary Structure Content**							**NRMSD**
	**α helix**	**α helix**	**β strand**	**β strand**	**Turns**	**Unordered**	**Total**	
	**(regular)**	**(distorted)**	**(regular)**	**(distorted)**				
0 min	-2%	1%	51%	25%	17%	3%	95%	0.018
10 min	-2%	2%	18%	13%	31%	37%	99%	0.035

The near-UV CD spectrum of human plasminogen (251–350 nm, Fig. 11) does not change considerably after 10 min of UV-illumination. The near-UV CD spectrum maximum of plasminogen remains constant with UV-illumination, at ~285 nm, region where mainly the protein aromatic residues absorb. Furthermore, after 10 min of illumination the ellipticity signal at 285 nm decreases only by 6%.

**Fig 12:**
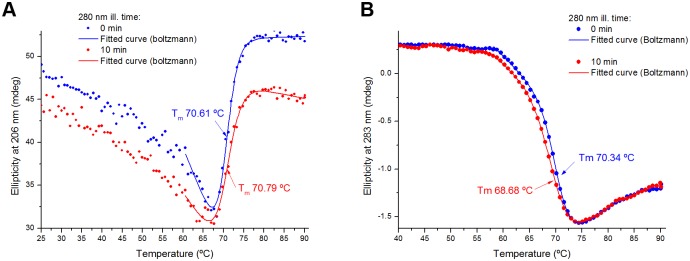
Circular dichroism thermal unfolding curves of human plasminogen. The circular dichroims thermal unfolding curves of a non-illuminated plasminogen sample (0 min 280 nm ill. time) or a UV illuminated plasminogen sample (10 min 280 nm ill. time) were obtained upon heating from 25 ºC to 90 ºC (1 ºC.min^-1^). A- The ellipticity signal was constantly monitored at 206 nm. B- The ellipticity signal was constantly monitored at 283 nm. Both curves were fitted using a modified Boltzmann function (see Table 7). The melting temperature of the protein, which corresponds to the transition mid-point of each curve, was recovered from the fitting (parameter *x*0) and is displayed for each sample.

**Circular dichroism based protein thermal unfolding studies**. The thermal unfolding curve of and the melting temperature of plasminogen samples were recovered using CD spectroscopy in order to see if the protein 3D fold is affected by brief UV illumination. The ellipticity intensity at 206 nm (far UV) and 283 nm (near UV) was continuously monitored from 25 ºC to 90ºC for a non-illuminated plasminogen sample (0 min 280 nm ill. time) and for an UV illuminated plasminogen sample (10 min 280 nm ill. time). The results are shown in Fig. 12A and 12B (206 nm and 283 nm fixed wavelength, respectively).

**Table 7:** Parameter values obtained upon fitting the circular dichroism thermal unfolding curves (ellipticity at 206 nm, Fig. 12A; ellipticity at 283 nm, Fig. 12B) of human plasminogen with a modified Boltzmann function.

**Fitting Equation**	$y=A2+B2.x+\frac{\left(A1+B1.x-A2+B2.x\right)}{\left(1+\exp\left(\frac{x-x0}{dx}\right)\right)}$				
**Ellipticity**		**206 nm**		**283 nm**	
**280 nm illumination time**		**0 min**	**10 min**	**0 min**	**10 min**
**Fitting Parameters**	R^2^	0.9936	0.99114	0.99973	0.99986
	*A1*	109.94 ± 8.03	77.37 ± 8.92	3.45 ± 0.12	2.84 ± 0.11
	*B1*	50.89 ± 2.88	53.75 ± 3.02	-4.47 ± 0.18	-4.66 ± 0.14
	*A2*	-1.18 ± 0.13	-0.72 ± 0.14	-0.05 ± 0.002	-0.05 ± 0.002
	*B2*	0.016 ± 0.034	-0.10 ± 0.04	0.04 ± 0.002	0.04 ± 0.002
	***x0***	**70.61 ± 0.14**	**70.79 ± 0.23**	**70.34 ± 0.05**	**69.6 ± 0.05**
	*dx*	1.36 ± 0.10	1.71 ± 0.16	1.43 ± 0.04	1.83 ± 0.04

Both non-illuminated and illuminated plasminogen samples show a similar thermal unfolding at 206 nm, with a rapid initial decrease in ellipticity intensity upon heating and a cooperative transition between 67–75 ºC. The ellipticity signal intensity of the illuminated sample is lower during the whole range (e.g. between 25 ºC and 60 ºC, this signal is in average 8.5% lower for illuminated plasminogen). A thermal transition is clearly visible in both samples. The curves were fitted using a modified Boltzmann model (fitted data in red and blue in Fig. 12A). The fitting results are displayed in Table 7. The melting temperature of the protein can be estimated from the temperature of mid-transition at 206 nm, corresponding to the fitting parameter *x*0. The estimated melting temperature (206 nm thermal denaturation curve) is almost the same for the non-illuminated and 10 min illuminated samples, 70.61 and 70.79 ºC, respectively.

Similarly, at 283 nm the thermal unfolding profile of plasminogen is not significantly affected by 10min of continuous 280 nm illumination (Fig. 12B). For both non-illuminated and illuminated samples there is a similar cooperative transition, occurring between 60 and 73 ºC, and there are no major differences between the two curves in ellipticity signal during the thermal heating of the protein (Fig. 12B). Fitting of the curves with a modified Boltzmann model (Fig. 12B and Table 7) shows that the estimated melting temperatures at 283 nm are also very similar for non-illuminated and illuminated plasminogen, of 70.34 and 69.98 ºC, respectively.

### Thiol group’s quantification

**Fig 13:**
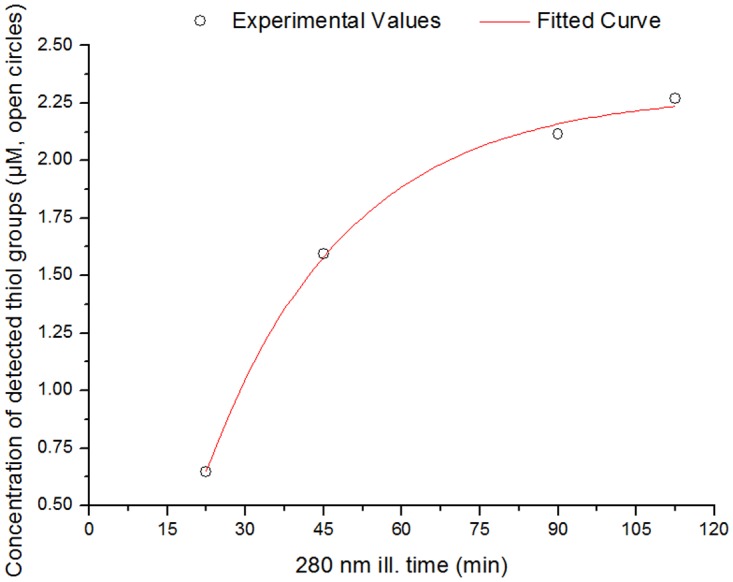
Increase in the concentration of detected free thiol groups (open circles) in human plasminogen UV-illumination. Detection of free thiol groups was carried out using the Ellman’s assay after 280 nm light continuous illumination (22.5 min, 45 min, 90 min, or 112.5 min) of human plasminogen in solution. The experimental values were fitted using an exponential function *y = y_0_ − A.e^-R0t^* (fitted curve in red), where *y* is the concentration of thiol groups (µM) at the 280 nm illumination time *t* (h), *y_0_* and *A* are constants and *R0* is the rate of thiol group formation (h^-1^). Fitted experimental parameters were: *y_0_* = 2.29 ± 0.07 µM, *A* = 3.78 ± 0.42 µM, *R0* = 0.037 ± 0.005 min^-1^. Root mean square error was 99.39%.

The concentration of solvent accessible thiol groups in human plasminogen has been determined with Ellman’s assay for a non-illuminated sample and for samples previously illuminated at 280 nm during 22.5–112.5 min (irradiance of 2.3 W.m^-2^). The concentration of free thiol groups increases exponentially with 280 nm continuous illumination (Fig. 13). After 112.5 min of illumination, the concentration of free thiol groups in human plasminogen is ~2.27 μM. Assuming that the formation of free thiol groups follows a first order kinetics (as indicates the 1^st^ order exponential model used) it is possible that more thiol groups are formed with increased 280 nm illumination time. The maximum value of thiol concentration (detected with the Ellmann’s reagent) can be estimated from the exponential model (*y = y_0_-A.e^-R0t^*) used for fitting and is given by *y_0_*, which is of 2.29 µM.

### Plasminogen UV activation

**Fig 14:**
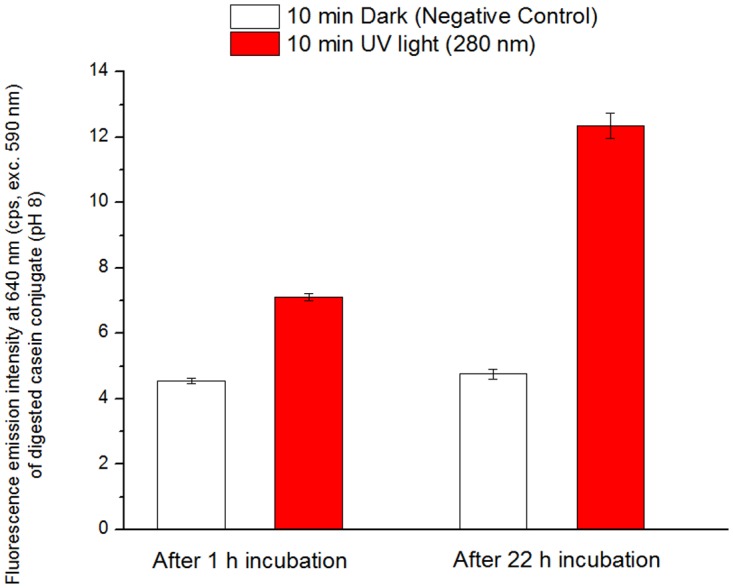
Fluorescence signal presented by the digested substrate for plasminogen kept in the dark (10 min in the dark, negative control) and UV illuminated plasminogen (10 min 280 nm illumination). Fluorescence signal is used as a measure of degradation of the substrate and to acess plasmin activity. The fluorescence readings were performed after 1 hour and 22 hours which is standard procedure for the used Casein-based substrate. The background fluorescence signal from substrate blank was subtracted.

The activity of illuminated (10 min, 280 nm) and non-illuminated (negative control) plasminogen was tested by measuring the fluorescence signal of the fluorescent cleaved casein-Bodipy products after 1 hour and 22 hours of incubation in the dark. It is a standard procedure using this particular substrate (Fig. 14). After 1 h of incubation the fluorescence emitted from the cleaved products by UV illuminated plasminogen is 1.6 fold larger than for non-illuminated plasminogen (negative control). At 22 h of incubation, the fluorescence emission intensity of cleaved products by UV illuminated plasminogen is 2.6 fold higher than for non-illuminated plasminogen (negative control), while the fluorescence signal for the non-illuminated plasminogen sample remains constant compared to the previous reading.

### Molecular Dynamics Simulations

**Fig 15:**
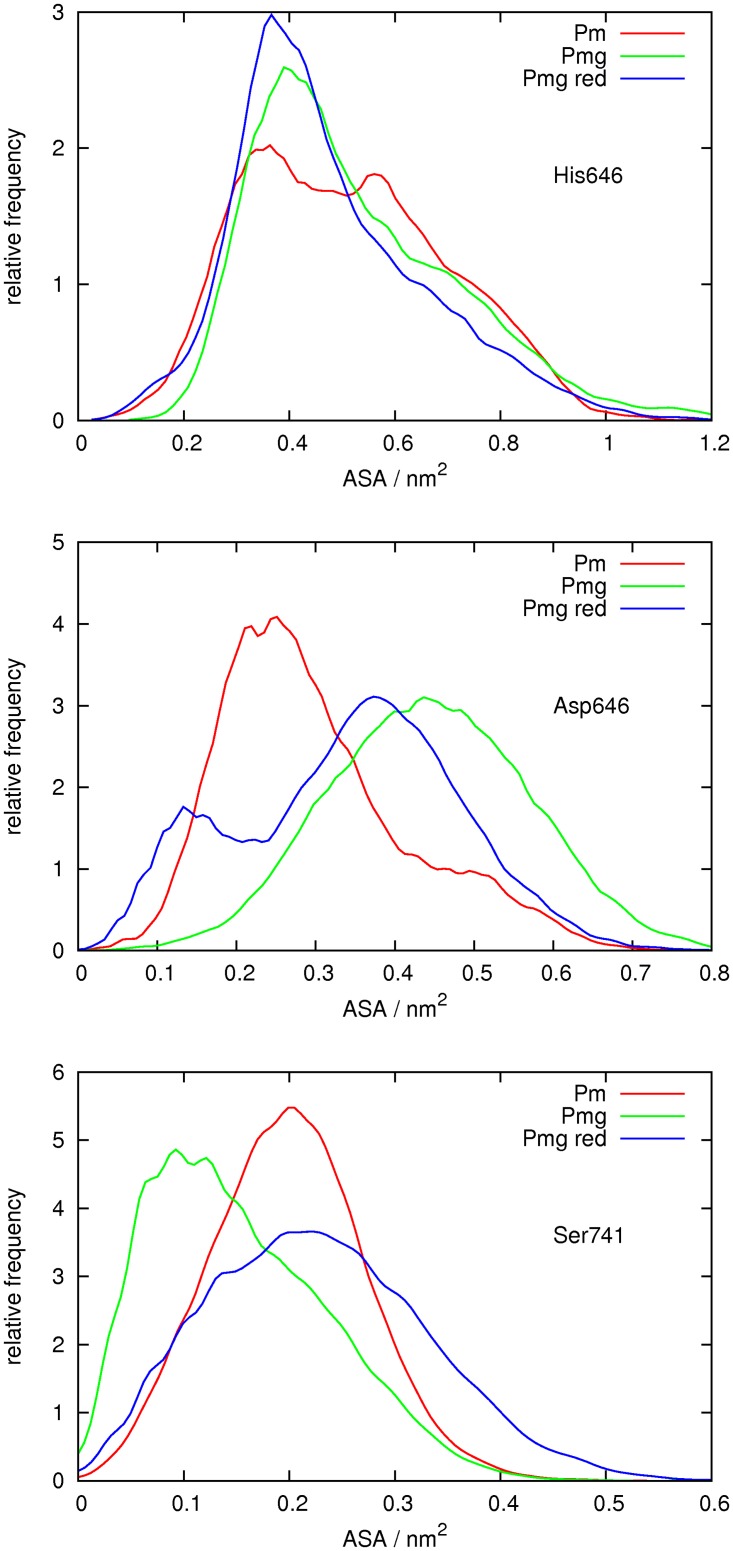
Distribution of solvent accessible surface area (ASA) of the catalytic triad. Frequency distributions of the ASA values of the side chains of His603 (top plot), Asp646 (middle plot) and Ser741 (bottom plot) obtained from the molecular dynamics simulations of plasmin (Pm) and of the catalytic domain of plasminogen in the native (Pmg) and reduced (Pmg red) forms. Each curve is normalized so that its integral is equal to 1.

The average structural trends of the catalytic domain of plasminogen are not largely affected by reduction of the Cys737-Cys765 bond. In particular, the loops 737–740 and 760–765 do not seem to exhibit significant changes that would make their average structure more similar to the ones in plasmin. Nonetheless, although no such average changes are observed, a significant reduction-induced increase of fluctuations is found in loop 760–765, the S1-entrance frame located close to the active site. This loop is the only segment that shows lower fluctuations in the native catalytic domain of plasminogen than in plasmin, which are recovered when the former is reduced. These fluctuations affect the range of solvent exposure of the catalytic triad, particularly of Asp646 and Ser741 (Fig. 15). While the exposure of His603 is essentially the same in all cases, one finds that, when compared to plasmin, Asp646 tend to be too exposed in the native catalytic domain of plasminogen, while the opposite is seen for Ser741. The effect of the reduction of the Cys737-Cys765 bond is, in both cases, to essentially bring back an exposure profile more similar to plasmin.

## Discussion

Human plasminogen is a complex molecule comprising seven folded domains. It contains a large number of aromatic residues, among which 19 Trp and 30 Tyr, which are present in all seven domains of the protein. The high number of aromatic residues makes this protein a likely target for photo-oxidation. Plasminogen architecture comprises 24 disulphide bonds, which have distinct functions within the protein, including e.g. stabilization of the kringle domains or restriction and recognition of the activation loop (see Introduction). For a protein of the size of plasminogen (769 amino acids), it is an exceptionally high number of disulphide bonds. It has an average fraction of disulphide bonds of 3%, compared to the expected 0.1% for a protein of that size (Fig. 4). Data displayed in Fig. 4 indicates that smaller proteins have usually higher average fraction of disulphide bonds values while the abundance of disulphide bonds is low in large proteins. A short protein has a hydrophobic core than a large protein and therefore depends to a larger extent on the structural stability provided by disulphide bridges. In plasminogen, disulphide bridges are ~30 times more abundant than expected for a protein of this length.

Most of these disulphide bonds have Trp or Tyr residues in close spatial proximity (≤ 8Å, see Results), which is a conserved structural feature in nature [31]. Additionally, in all the domains there is at least one aromatic residue less than 6 Å away from a disulphide bond (See Results), therefore close to the van der Waals contact distance between atoms (≤ 5.2 Å, [59]. This makes these disulphide bonds excellent targets for photo-induced cleavage [22]. Their proximity to aromatic residues favours the transfer of electrons formed upon upon UV excitation of aromatic residues (see Introduction) to the disulphide bonds. Hence, it is expected that several of the protein disulphide bonds are disrupted upon UV illumination, inducing conformational changes that can affect the functionality of the biomolecule.

### Tryptophan and Tyrosine photobleaching

Prolonged UV illumination of plasminogen leads to decreasing amounts of intact Trp and Tyr molecules. A decrease in both excitation and emission intensity of Trp and Tyr (losses in intensity ~10–11%, Fig. 5, Fig. 6A and Fig. 8) has been observed upon 280 nm illumination (45 min). This suggests that these aromatic residues are progressively photo-bleached. Photobleaching of the aromatics can imply the formation of new species, Trp and Tyr derivatives (see Fig. 3), and the triggering of other photochemical and photophysical mechanisms, which will now be discussed.

### Dityrosine and other tyrosine derivatives

A new species that strongly emits at 396 nm (exc. 325 nm, Fig. 7) is progressively formed upon 280 nm illumination of plasminogen. These spectral features are consistent with the fluorescence characteristics of dityrosine (Em^max^ at 400–409 nm, see Table 1). The emission intensity increase at 396 nm is correlated with an increase in excitation intensity (em. 405 nm, Fig. 9) at 2 separate peaks located at ~283 nm and ~321 nm. These wavelengths are in agreement with the absorption maxima of dityrosine ionic species at 283 nm (deprotonated form) and 315 nm (protonated form) [61, 62], Table 1). In fact, we also observed an increase in the intensity at ~375–450 nm in the emission spectra recorded with excitation at 280 nm and 295 nm (Figs. 6A, 8). The formation of this new fluorescent species was monitored during 280 nm illumination by following the kinetics of fluorescence emission at 405 nm at the two relevant excitation wavelengths: 280 nm and 325 nm (Figs. 6 and 7, respectively). Both curves followed single exponential kinetics and had matching values for the kinetic constant *k* (fluorescence exponential emission increase) (Table 5). This indicates that at both excitation wavelengths (280 nm and 325 nm) we are monitoring the formation of the same fluorescent species via a first order reaction, which matches the mechanisms of dityrosine formation [30].

Dityrosine formation has been previously observed upon exposure of proteins to UV-light, e.g. in calmodulin [37, 63], elastine hydrolysates [64], and insulin [30]. While in solution dityrosine emits at 407–409 nm, it has been observed that in proteins dityrosine emission is blue-shifted, being centered ~400 nm. This is true for calmodulin and insulin and is consistent with our observations in this work (em_max_ at 396 nm, Fig. 7). The close spatial proximity between Tyr residues in plasminogen favours dityrosine formation (see Table 4). Furthermore, we have documented that three or even four Tyr residues are in favourable distance for Tyr cross-linking (Table 4), which could result in more than one Tyr cross-linking within the same chain. Pulcherosine and Trityrosine, which are trimers of Tyr formed upon Tyr radical cross-linking, have similar fluorescence characteristics compared to dityrosine (Table 1). Thus, these specii could also contribute to the observed emission intensity increase at 396 nm.

### NFK and Kyn

Dityrosine is not the only species that can contribute to the strong increase in emission intensity at ~396 nm (exc. 325 nm, Fig. 7) observed upon UV illumination of plasminogen. NFK, a Trp derivative (Fig. 3), can fluoresces between 400 nm and 440 nm, depending on pH, neighbouring functional groups and solvent polarity (Table 1 and [38]). Fluorescence emission of the two formed species upon 280 nm illumination, NFK (Em^max^ between 400–440 nm) and dityrosine (Em^max^ at 400–409 nm) might overlap in the spectra obtained upon 325 nm excitation (Fig. 7). The same is true for the excitation spectra obtained with emission fixed at 405 nm (Fig. 9) and 434 nm (Fig. 10), where the excitation peaks found at 283–286 nm and 327–326 nm might be resultant of dityrosine and NFK excitation. Absorption and excitation of NFK are also strongly dependent on experimental conditions. For instance, in solution at pH 7.4 NFK excitation peaks are centred at 265 nm and 330 nm, while at pH 11 these are centred at 240 nm and 315 nm (Table 1, [65]).

It is not likely that Kyn (another Trp derivative, see Introduction and Table 1) is formed upon prolonged UV illumination of human plasminogen. We did not detect any Kyn typical fluorescence during the fluorescence measurements that were carried out in this work (data not shown, emission spectra recorded with 365 nm excitation, and excitation spectra obtained with emission fixed at 480 nm).

### Disulphide Bond Disruption

UV illumination of Trp and Tyr also leads to increasing amounts of free SH groups detected with the Ellman’s assay (Fig. 13). The close spatial proximity of aromatic residues and SS bonds in plasminogen favors the breakage of disulphide bonds upon UV illumination of neighboring aromatic residues. The proximity to disulphide bonds allows direct electron transfer from the aromatic residues to disulphide bonds (see Introduction, schemes 2 and 3). Photoionization of Trp and Tyr and subsequent formation of solvated e^-^_aq_ may also be a mechanism involved in the photo induced breakage of disulphide bonds (See Introduction, schemes 1 and 3).

The formation of free disulphide groups upon continuous 280 nm illumination of plasminogen follows a single exponential kinetic. It indicates that the breakage of disulphide bonds is consistent with a first order reaction. The maximum value for the concentration of free and solvent accessible SH groups is 2.29 μM. Considering that the protein concentration used for the Ellman’s assay experiments was 0.97 µM, that plasminogen has 24 disulphide bonds, and that 1 SH group is formed per molecules bond disrupted (the other S atom is in a radical from), the maximum concentration of free thiol groups that could be present would be of 23.3 µM. Thus, we can conclude that at least two disulphide bonds have been disrupted in each plasminogen molecule after 280 nm illumination (Fig. 12). The number of disulphide bonds broken may be higher than estimated by the Ellman’s assay. It has been reported that due to steric or electrostatic constraints, some protein sulfhydryls do not react with DTNB [66–68]. In fact, most of the cysteine residues involved in disulphide bonds in plasminogen have low ASA values (less than 12% of the total Cys ASA, see Results) and it is not certain that the most solvent acessible disulphide bonds (7 of them have more than 31% of total Cys ASA, see Results) are all cleaved upon UV-illumination.

### UV light effects on secondary and tertiary structure of plasminogen

The chemical modifications on Trp, Tyr residues and the breakage of disulphide bonds that occur upon continuous UV exposure of plasminogen are likely to affect the native three-dimensional fold of the protein. The disulphide bonds of plasminogen have important structural roles (e.g. stabilization of the kringle domains, restriction and recognition of the activation loop etc). Furthermore, dimerization of Tyr residues (forming DT) can lead to intra- or inter-molecular cross-linking in proteins [37, 69, 70]. The possible conversion of Trp to NFK can also lead to protein structural damage, since NFK is also a photosensitizer that can generate reactive oxygen species (ROS) upon UV light absorption [71]. The fluorescent photo derivatives of Trp and Tyr are formed quickly upon UV illumination (Figs. 6B and 7 insert). If we consider the fluorescence emission kinetics (em. 405 nm) recovered on Figs. 6B and 7 as indicatives of DT and/or NFK formation we can estimate that after only 10 min of UV illumination ~56% of the total possible DT and/or NFK products are already formed.

The impact of UV illumination on the secondary and tertiary structure of human plasminogen was evaluated using CD spectroscopy. We have observed a decrease in far UV CD ellipticity at 206 nm (33.4%) and a 3 nm red-shift of the spectrum maximum after only 10 min of 280 nm illumination (Fig. 11). This is consistent with the loss of protein secondary structure contents upon UV-illumination.

The secondary structural content of the protein was estimated upon deconvolution of the far UV CD spectra with the CD. This analysis shows that non illuminated plasminogen contains a large fraction of β strands (76%) and turns (17%), and almost no helical content (below 1%) (Table 6). This numbers are in agreement with the structural motifs observed in the 3D crystal structure of native human plasminogen, confirming that the deconvolution of the spectra worked well. Promotif analysis of the crystal structure (recovered with PDBsum, http://www.ebi.ac.uk/pdbsum/, input file: 4A5T.pdb, full-length native human plasminogen, 3.49 Å resolution [41]) shows that the native human plasminogen secondary structure is mostly constituted by strands (19.3%) and other types of structure (73.9%), including a great number of β and gamma turns (130 in total). Furthermore, the helical content of the protein is low (6.7% of the structure).

Deconvolution data (Table 6) indicates that the β strand content of human plasminogen is mostly disarranged upon UV-illumination, resulting in an increase in unordered secondary structure. The results also show a slight increase in turn content with UV illumination (17% to 31%), probably due to disarrangement of β strands.

There is no doubt that UV illumination leads to losses in secondary structural features of plasminogen. However, it is not likely that these structural changes will affect tremendously the overall native fold of the protein. Human plasminogen retains a cooperative thermal unfolding ellipticity curve at 206 nm even if it is previously exposed to 280nm for a short illumination period (Fig. 12A). Furthermore, the melting temperature recovered from the curves is similar for non-illuminated and illuminated plasminogen: 70.61 ºC and 70.79 ºC respectively (Table 7).

Similarly, 10 min 280nm illumination did not lead to significant changes in the near UV CD features of plasminogen. The near UV CD spectra of non-illuminated and illuminated plasminogen are both centered at ~285 nm, where Tyr and Trp residues contribute. Most of the the CD signal of plasminogen at 285 nm (~94%) is retained after 10 min of UV illumination. Additionally, the near UV (283 nm) CD thermal unfolding of human plasminogen is practically the same before and after 10 min of UV illumination. The two samples had similar cooperative thermal unfolding curves (Fig. 12B) and concordant melting temperature values (70.34 ºC and 69.68 ºC before and after UV illumination, respectively, Table 7). The data indicates that the local tertiary environment of Tyr and Trp residues does not change considerably. This is supported by the Trp fluorescence data (Fig. 8). There is no change in the wavelength of maximum emission (332 nm) with 280 nm illumination, indicating no changes in solvent accessibility and thus in the environment of the Trp residues.

### UV light Activation of Plasminogen

Brief UV illumination of plasminogen (10 min, 280 nm) results in an increase of detected cleaved enzyme substrate (2.6 fold increase in fluorescence emission for 22 h of substrate incubation, Fig. 14). It shows that the enzymatic activity of plasminogen is enhanced upon brief UV illumination and it indicates that the proenzyme can be activated by UV exposure. The fact that the fluorescence signal of the non-illuminated sample does not increase between these measurements, and that UV activated plasminogen displays significant larger substrate degradation at both readings, indicates that plasminogen is activated by brief 280nm illumination and that no or little activity is displayed by the non-activated plasminogen (negative control).

We have observed that the overall fold of the protein is not compromised after exposing the protein to a low UV-light dosage (CD studies, 10 min illumination at 280 nm, 2.1 W.m^-2^). It is also relevant to mention that the irradiance at 280nm used in our present study (Fluorescence studies and Ellman’s assay: 2.3 W.m^-2^, CD studies 2.1 W.m^-2^) is in the same order of magnitude than the total irradiance of sunlight in the UVB region (280–315 nm), which is reported to be 0.78 W (annual average value based on the data from ASTM G173–03 Air Mass 1.5 Reference Spectra, American Society for Testing and Materials (ASTM) [30]).The used UVB dose proved to be the sufficient to induce limited conformational changes that favour plasminogen activation, despite not affecting the overall conformation of the protein. 280nm illumination of Trp and Tyr residues in human plasminogen leads to the breakage of two or more disulphide bonds. If the breakage of these disulphide bonds occurs within the serine protease domain it is likely that conformational changes occur inside and around the active site upon UV illumination. Two disulphide bonds are particularly relevant for activation of plasminogen to plasmin: Cys558-Cys566 and Cys737-Cys765. Cys558-Cys566 stabilizes the activation loop (Fig. 1B) and it has been proposed that it has a role in its recognition by uPA, tPA and fibrin [72]. Furthermore, it has been proposed that the disulphide bond Cys558-Cys566 may be important for the enzymatic specificity of plasminogen [4]. Trp573 is 9.06 Å away from this disulphide bond, and it could mediate disruption of this disulphide bond. However, there are other Trp and Tyr residues in closer proximity to other disulphide bonds in the catalytic domain that could be more likely targets for photolysis (Table 3). Furthermore, it has been proven that the activity of plasmin does not depend on the presence of the Cys558-Cys566 disulphide bond [72].

Cys737-Cys765 is the only disulphide bond in the serine protease domain that has an aromatic residue within van der Waals distance (Trp761 at 4.24 Å) (Fig. 1B), which makes it the most likely target of photolysis upon Trp excitation. It has also a Tyr residue in close proximity (Tyr 774, 8.35 Å away). Additionally, its pair of cysteine residues is one of the most solvent accessible in native human plasminogen and the most solvent accessible in the serine protease domain (ASA_Cys737_ = 57Å^2^; ASA_Cys765_ = 21Å^2^; see S1 Table). This makes this Cys residues good acceptors of solvated electrons generated upon aromatic excitation. Cys737-Cys765 and Cys680–747 have an unique bond geometry among the disulphide bonds in plasminogen called –RHStaple [18]. As previously mentioned, this geometry is typical of allosteric disulphide bonds, known for controlling protein function by mediating conformational change via their reduction or oxidation [18]. Chen and Hogg report that one or more of the plasminogen disulphide bonds are redox-active [18]. Additionally, one of these redox-active disulphide bonds may be important in the mechanism of release of angiostatin, a fragment of plasmin and the precursor for an inhibitor of angiogenesis. It occurs upon reduction of the disulphide bonds in K5 (kringle 5) [18]. As previously mentioned Cys737-Cys765 holds together two important loops for plasminogen activation: the oxyanion stabilizing loop (residues 737–740) and the S1 entrance-frame (residues 760–765). These loops are located in a region close to the activation pocket and that suffers conformational changes upon activation. It is possible that the likely disruption of this disulphide bond induces a similar conformational change in plasminogen, rendering the catalytic site active (Fig. 2). Several allosteric disulphide bonds have been identified in proteins involved in thrombosis and thrombolysis. In tissue factor, an important protein for the initiation of blood coagulation, it has been proven that the cleavage and formation of a particular allosteric disulphide bond (Cys186-Cys209) is crucial for changing between the different functions of this co-factor [73]. It is possible that in plasminogen/plasmin Cys737-Cys765 has a similar functional role, and depending on its redox state, induces the switch between inactive (plasminogen) and active (plasmin) protein. This is indeed supported by the molecular dynamics simulations, which reveal reduction-induced changes of the solvent exposure of the catalytic triad (Fig. 15). Compared to plasmin, the native catalytic domain of plasminogen exhibits two features which may explain its inactivity: (1) a more exposed Asp646, which role in stabilizing the transiently charged His during the initial reaction steps may be compromised; (2) a too buried Ser741, which may lose its ability to complex with the substrate. Reduction of the Cys737-Cys765 bond largely restores the exposure profiles, which may explain the activation of plasminogen by UV light. Interestingly, this restoring of solvent exposure is associated with increased fluctuations of the S1-entrance frame (loop 760–765), which may impart the flexibility/plasticity necessary for structural rearrangements of the catalytic triad. Structural flexibility seems to be a key factor in catalysis, as strikingly illustrated by enzymes in organic solvents, where the protein becomes essentially rigid, requiring a minimum amount of water to regain flexibility and catalytic function [74, 75]. Analogously, the Cys737-Cys765 bond in the native catalytic domain of plasminogen may cause the active site region to lose the plasticity required for catalysis, which would be restored upon reduction.

### UV light Activation of Plasminogen versus UV deactivation of insulin

The present study will now be compared with our previous study on the effect of UV light on insulin [30]. UV illumination of plasminogen at irradiance levels in the same order of magnitude of the total irradiance of sunlight in the UVB region for a short period of time (10 minutes) leads to the activation of plasminogen into plasmin. The previous insulin study reports the UV induced biological deactivation of the protein [30]. The UV irradiance level used was the same but the illumination times used in the insulin study were longer, reaching a few hours. These two papers complement each other since one paper shows that UV light can be beneficial in activating a key medical protein and the other paper shows that UV deactivates a key medical proteins such as insulin, a negative effect. The UV induced changes depend on the protein and on the illumination conditions.

Insulin is an obvious candidate for UVB induced deactivation. Human insulin is made of two chains linked together by two inter-chain SS (CysA7-CysB7 and CysA20- CysA19) and an additional SS connects CysA6 and CysA11 within the A chain [30]. Since UVB excitation of the protein’s aromatic residues leads to the disruption of disulphide bridges in proteins and the structure of insulin is so dependent on these covalent bonds, it is expected that UVB light will break the unique bonds that link the 2 chains. The result is the destruction of the insulin structure and loss of activity. UVB excitation of insulin also leads to dityrosine formation and insulin dimerization via dityrosine cross-linking. In the insulin dimer the classical binding region would not be available for recognition by the insulin receptor, resulting in permanent loss of function, as observed in our previous study.

The present paper, on the other hand, shows a beneficial effect of UVB illumination of plasminogen at short illumination times, i.e., its activation into plasmin. We propose that photonic activation occurs upon photo-cleavage of a functional allosteric disulphide bond, Cys737-Cys765, which induces change in the solvent accessibility and plasticity of the active site (see Fig. 15). A protein structure such as plasminogen is a likely candidate for UV induced activation due to the presence of an allosteric disulphide bridge that holds together 2 loops that need to be displaced in order to render the protein active. It is most likely that proteins containing allosteric disulphide bridges are interesting candidates for UV induced activation. Our study concurs with the view that disulfide bonds have been added to proteins not only to help hold them together, but also as a way of controlling how they work [76]. Cleavage of disulfide bonds is emerging as an important mechanism of protein control in the blood circulation [77].

The presented results show that plasminogen can be activated by low dose 280nm radiation. Possible applications of UV induced activation of plasminogen are numerous—a few are mentioned below:

- Activation of plasminogen using plasminogen activators is used in clinical practice for treatment of patients with various vascular diseases. For situations where activation and/or enhanced activation of plasminogen is needed, e.g. for clinical treatment of strokes, low dose UV activation could be beneficial compared to infusion of the common (and expensive) plasminogen activator proteins such as tPa or uPA.
- If plasminogen activation is inhibited due to a high amounts of tPA and/or uPA inhibitors therapies based on infusions of these activators will not be effective for plasminogen activation. In contrast, plasminogen activation via UV light would not be affected by the presence of tPA/uPA inhibitors.
- Catheters/tubes for draining blood/plasma e.g. from lung tissues often clot (Stevens and Tobias, 2001). Introducing UV activation locally through these catheters, e.g. by optical fibres in the catheter construction or by constructing the catheter in a UV guiding material and introduce UV light may reduce this critical problem.

The dangers of overexposure to sunlight have been well publicized, and there is reluctance for the use of UV light in medical therapy. However, UV light has already been used to successfully treat a number of diseases, including rickets, psoriasis, lupus vulgaris, vitiligo, atopic dermatitis and localized scleroderma and jaundice [78]. The effects of UV light on biomolecules, cells and tissues will depend on the energy delivered per unit area. Furthermore, it will also depend greatly on the wavelength that is used. Skin cancer is the result of exposure to both UVB and UVA (315–400 nm) radiation upon acute overdosing (causing sunburn) and lifelong cumulative exposure [79]. Melanoma is most likely to be caused by UVA, while the UVB fraction of the solar radiation has mostly benign effects such as erythema, melanogenesis (melanin production), vitamin D synthesis, and non-melanoma skin cancer [80]. The health effects of UV exposure are dose and wavelength dependent. It is our hope that the presented results will inspire the development of novel solutions based on UV activation of plasminogen that could help patients with relevant diseases.

## Supporting Information

S1 Table Characteristics of the disulphide bonds in human plasminogen. The solvent-accessible surface area (ASA) of the cysteine residues, classification and the protein domain are described for each of the disulphide bonds of the full-length native human plasminogen.
(DOCX)
