# Correction: Photonic Activation of Plasminogen Induced by Low Dose UVB

**DOI:** 10.1371/journal.pone.0144794

**Published:** 2015-12-11

**Authors:** Manuel Correia, Torben Snabe, Viruthachalam Thiagarajan, Steffen Bjørn Petersen, Sara R. R. Campos, António M. Baptista, Maria Teresa Neves-Petersen

An affiliation for the 7^th^ author is not included. Maria Teresa Neves-Petersen is affiliated with: **2** BioPhotonics Group, Department of Nanomedicine, International Iberian Nanotechnology Laboratory (INL), Braga, Portugal and **7** Department of Clinical Medicine, Aalborg University, Søndre Skovvej 15, 9000 Aalborg, Denmark.
