# Supplementary material for: Photonic Activation of Plasminogen Induced by Low Dose UVB
Source: PLoS One. 2015 Jan 30;10(1):e0116737. doi: 10.1371/journal.pone.0116737 (PMC4312030; doi:10.1371/journal.pone.0116737)
Supplement: S1 Table — The solvent-accessible surface area (ASA) of the cysteine residues, classification and the protein domain are described for each of the disulphide bonds of the full-length native human plasminogen. (DOCX) [file pone.0116737.s001.docx]

| **Disulphide Bond (Cys1-Cys2)** | **Cys1 ASA (Å^2^)** | **Cys2 ASA (Å^2^)** | **Disulphide Bond Classification** | **Protein Domain** |
| --- | --- | --- | --- | --- |
| C30-C54 | 0 | 0 | .+/-RHSpiral | AP |
| C710-C726 | 0 | 0 | .-LHSpiral | SP |
| C215-C238 | 0 | 0 | .-LHSpiral | K2 |
| C105-C145 | 1 | 0 | .+/-LHSpiral | K1 |
| C277-C316 | 1 | 0 | .+/-LHSpiral | K3 |
| C680-C747 | 0 | 2 | **.-RHStaple** | SP |
| C34-C42 | 0 | 3 | .+LHHook | AP |
| C379-C418 | 3 | 0 | .+/-LHSpiral | K4 |
| C483-C524 | 3 | 0 | .+/-LHSpiral | K5 |
| C187-C226 | 5 | 0 | .+/-LHSpiral | K2 |
| C588-C604 | 3 | 5 | .-LHSpiral | SP |
| C305-C328 | 0 | 6 | .-LHSpiral | K3 |
| C512-C536 | 0 | 6 | .-LHSpiral | K5 |
| C407-C430 | 0 | 7 | .-RHHook | K4 |
| C133-C157 | 0 | 12 | .-LHSpiral | K1 |
| C462-C541 | 21 | 24 | .-/+RHHook | K5 |
| C166-C243 | 9 | 26 | .+/-LHStaple | K2 |
| ***C558-C566*** | 1 | 32 | .-RHSpiral | SP |
| C256-C333 | 35 | 27 | .-/+RHHook | K3 |
| C84-C162 | 42 | 3 | .+/-RHSpiral | K1 |
| C169-C297 | 21 | 49 | .-LHHook | K2-K3 |
| C548-C666 | 54 | 8 | .-/+RHHook | SP |
| ***C737-C765*** | 57 | 21 | **.-RHStaple** | SP |
| C358-C435 | 41 | 70 | .-/+RHHook | K4 |

Table S1. Characteristics of the disulphide bonds in human plasminogen. The solvent-accessible surface area (ASA) of the cysteine residues, classification and the protein domain are described for each of the disulphide bonds of the full-length native human plasminogen.

Disulphide bonds with –RHStaple geometry and important for plasminogen enzymatic activation are highlighted in bold and italic, respectively.
